# Implementation of advance care planning decision aids for patients undergoing high-risk surgery: a field-testing study

**DOI:** 10.1186/s12904-022-01068-2

**Published:** 2022-10-12

**Authors:** Kanako Yamamoto, Toshimi Kaido, Tadao Yokoi, Gen Shimada, Takashi Taketa, Kazuhiro Nakayama

**Affiliations:** 1 Department of Critical Care Nursing, Graduate School of Nursing Science, St. Luke’s International University, Tokyo, Japan; 2 Department of Nursing Informatics, Graduate School of Nursing Science, St. Luke’s International University, Tokyo, Japan; 3 Department of Gastroenterological and General Surgery, St. Luke’s International Hospital, Tokyo, Japan

**Keywords:** Advance care planning, Critical care, Surgery, Decision aid, Patient, Family, Decision-making, Intensive care unit, Implementation, Sheard decision making

## Abstract

**Background:**

Patients undergoing high-risk surgery are at a risk of sudden deterioration of their health. This study aimed to examine the feasibility of the development of two patient decision aids (PtDAs) to assist patients undergoing high-risk surgeries in informed decision-making about their medical care in a crisis.

**Methods:**

This field testing implemented two PtDAs that met the international criteria developed by the researchers for patients before surgery. Study participants were patients scheduled to be admitted to the intensive care unit after surgery at one acute care hospital in Japan and their families. The study used a mixed-methods approach. The primary outcome was patients’ decision satisfaction evaluated by the SURE test. Secondary outcomes were the perception of the need to discuss advance care planning (ACP) before surgery and mental health status. The families were also surveyed on their confidence in proxy decision-making (NRS: 0–10, quantitative data). In addition, interviews were conducted after discharge to assess the acceptability of PtDAs. Data were collected before (preoperative outpatients, baseline: T0) and after providing PtDAs (in the hospital: T1) and following discharge (T2, T3).

**Results:**

Nine patients were enrolled, of whom seven agreed to participate (including their families). The SURE test scores (mean ± SD) were 2.1 ± 1.2 (T0), 3.4 ± 0.8 (T2), and 3.9 ± 0.4 (T3). The need to discuss ACP before surgery was 8.7 ± 1.3 (T1) and 9.1 ± 0.9 (T2). The degree of confidence in family surrogate decision-making was 6.1 ± 2.5 (T0), 7.7 ± 1.4 (T1), and 8.1 ± 1.5 (T2). The patients reported that using PtDAs provided an opportunity to share their thoughts with their families and inspired them to start mapping their life plans. Additionally, patients wanted to share and discuss their decision-making process with medical professionals after the surgery.

**Conclusions:**

PtDAs supporting ACP in patients undergoing high-risk surgery were developed, evaluated, and accepted. However, they did not involve any discussion of patients’ ACP treatment wishes with their families. Medical providers should be coached to provide adequate support to patients. In the future, larger studies evaluating the effectiveness of PtDAs are necessary.

**Supplementary Information:**

The online version contains supplementary material available at 10.1186/s12904-022-01068-2.

## Background

Patients undergoing high-risk surgery may face a life-threatening crisis. Shared decision-making (SDM) is essential for physicians and patients, allowing the discussion of the risk of the operation [1–3]. Patient decision aid (PtDA) is an intervention that promotes SDM and improves the quality of patient decision-making [4]. PtDAs are tools designed to help patients participate in decisions regarding health care. PtDAs provide information on treatment options, including benefits and risks, and help clarify patient values [5, 6].

Surgery is a type of proactive treatment, which involves a complicated decision-making process in emergencies. It is important for patients, their families, and healthcare providers to discuss the risks of anesthesia and surgery in advance, but this practice remains challenging [7]. Despite this, consideration of end-of-life care for perioperative patients has historically been a taboo among surgeons [8]. In addition, there is a fear that patients may not receive appropriate lifesaving treatment if they express their wishes for end-of-life care and inform physicians before surgery [9]. While ensuring that patients receive appropriate life-saving treatment, they are entitled to medical care with confidence, and their autonomy should always be respected, so that they do not receive inappropriate treatment or continue to live in an unwilling state. However, the effect of PtDAs on advance care planning (ACP) support in patients undergoing high-risk surgery has not yet been clarified.

PtDA is crucial when a when the decision-making process is difficult due to the availability of multiple options [5]. A key point of PtDAs is that the factors that influence decision-making have values and support, clarifying values in decision support [5, 10]. ACP is defined as the ability of an individual to define goals and preferences for future medical treatment and care by discussing these with their families and health care providers, and recording and reviewing these preferences as needed [11, 12]. PtDAs help patients make decisions regarding the medical care they want to receive, including the critical situations requiring ACP, and can help them reflect and become aware of their thoughts and values.

Previous studies on ACP and advance directives (ADs) in perioperative patients faced challenges as the participants often felt anxious or refused to participate because they did not recognize the need [13–16]. A study of patients admitted to the intensive care unit and their families reported that providing information about cardiopulmonary resuscitation and preferences for resuscitation treatment during hospitalization did not increase anxiety [17]; however, clinical evidence to support this fact is inadequate [18]. In a previous study on ACP support for preoperative patients, 80% of the medical professionals were concerned about an increase in patients' anxiety; however, only 20% of the patients reported that ACP support before surgery resulted in anxiety and discomfort [19]. Preoperative ACP support cannot be initiated without the consent of the physician. Therefore, it is necessary to prove that preoperative ACP support is not harmful to patients and their families. Since discussion between healthcare providers, patients, and their families is crucial in ACP, its implementation and acceptability need to be determined in a clinical setting. In addition, it has recently been pointed out that ACP support does not improve the quality of end-of-life care and does not have the expected benefit [20]. Therefore, it is also necessary to explore whether it is possible to step up to clinical studies to evaluate the outcome indicators and validity of ACP support for high-risk surgical patients.

This study aimed to develop PtDAs for ACP to be implemented before the operation for patients, who were scheduled for high-risk surgery. The feasibility and acceptability of PtDAs were evaluated.

## Methods

### Study participants, setting, and design

Patients scheduled to undergo gastrointestinal surgery and thereafter scheduled for admission to the intensive care unit, as well as their families, were included in this study. The eligibility criteria were as follows: age ≥ 20 years, able to communicate, received permission from the attending physician, and decided to undergo surgery. The exclusion criteria included the patients with a history of dementia, patients with current cognitive decline, and patients requiring emergency surgery. The participants' families were selected from those who were designated by the patient and whose consent was obtained. The study was designed to conduct a feasibility study using PtDAs for ACP. Although there is no clear definition of a feasibility study [21–23], it includes the assessment of the possibility of conducting a study that includes an RCT [21, 22] or the assessment of essential parameters for a study [23]. The appropriate sample size for the feasibility study is also not defined. In the previous study, the minimum sample size for the feasibility study was around 10 [24], and the research method literature described it as around 5–10 [25]. Although the sample size was minimal, the main purpose of this study was not to evaluate outcomes but to investigate the possibility and process of clinical implementation of the developed PtDAs. Further, because it is important that patients voluntarily participate in ACP, this study was conducted in a single arm without a comparison group.

This study was conducted in an acute hospital with 500 beds in Japan from August to November 2021. ACP support for preoperative patients had not been previously conducted in the hospital. This was an intervention study using newly developed PtDAs for ACP for patients undergoing high-risk surgery. To evaluate the effectiveness of the PtDA, it was necessary to improve the quality of patients’ decision-making and clarify how patients used the PtDAs to improve their decision-making. The implementation study assessed whether patients can initiate voluntary ACP behavior with the help of PtDAs alone. For this reason, this study adopted the explanatory sequential design of a mixed method in which qualitative and quantitative data were collected and interpreted [26, 27]. The first step is to analyze the quantitative data, and the second is to analyze the qualitative data to understand the results further. The combined interpretation of the findings from these two steps is intended to integrate quantitative and qualitative research approaches.

### Patient decision aid

The PtDAs used for this study were developed following a systematic model development process [28] (Additional file 1) by the researchers, based on a survey of the needs of patients, their families, and healthcare workers in the context of high-risk surgeries [9, 29]. The Ottawa decision support framework conceptualizes the support that patients, families, and healthcare providers need for difficult decisions [29, 30]. The framework assesses the patient’s decision support needs using PtDAs to determine its impact on the outcome of the patient's decision, including its quality and the process itself. PtDAs developed from this framework have improved the quality of patient care, including the decision-making processes, compared to conventional care [1]. The PtDA developed by the researchers met this conceptual framework and the criteria established by the International Patient Decision Aid Standard [31, 32] (Qualifying criteria 6/6, Certification criteria 38/40).

The configuration of the PtDAs is shown in Table 1. PtDA_A determines whether a patient considers a treatment preference and communicates it to a surrogate decision-maker or health care provider. PtDA_B determines whether a patient decides to continue or stop treatment with the hope of prolonging life if recovery becomes difficult. Two PtDA made it possible for patients to consider ACP at their pace by considering the medical treatment they would like to receive and discussing it with a medical provider and focusing on treatment options if they deteriorate suddenly. The two PtDAs were paper-based booklets that patients could write on. It was explained to the patients that if they want to talk to their family or medical provider, the PtDAs could be used to take steps to discuss the ACP (Additional file 2).

**Table 1:** Content of patient decision aids used in this study

Chapter	Contents	Setting
PtDA_A		
Total number of pages	20	
Option	1 Do not communicate your ACP’s wishes to surrogate decision-makers and health care providers	
	2 Communicate your ACP's wishes to surrogate decision-makers and health care practitioners	
Step 1	・Description of the situation and treatment in the event of life crisis	Read
Step 2	ACP steps	Read
	About surrogate decision-makers	Read and write
	About the discretion	Read and check
	Writing down the patient’s values	Check and write
Step 3	Advantages and disadvantages of options	
	1 The patient’s wishes are always reflected in the treatment	Read
	2 Consideration of ACP may increase preoperative anxiety	
	3 Augmenting the surrogate decision-maker’s anxiety by talking about ACP to patients before surgery	
	4 Mental burden on surrogate decision-makers in making proxy decisions	
	5 Change in confidence of surrogate decision-makers with and without prior ACP discussions	
Step 4	Value clarification exercise	Check
Step 5	Guidance in decision-making and decision	Check
Supplement	Voice of ICU survival	Read
	Voice of healthcare providers	
PtDA_B		
Total number of pages	14	
Option	1 Continue to receive all treatment regardless of survival rate	
	2 Discontinue life-sustaining treatment when the survival rate decreases	
Step 1	How do you consider your hopes and wishes for life-sustaining treatment?	Read
Step 2	Physical conditions that reduce survival rates (each organ)	Read
	Treatment in ICUs (ventilators, assisted circulatory devices, dialysis, sedatives and analgesics, vasopressor, blood transfusion, and nutrition)	
	Withdrawal/withholds life-prolonging treatment	
Step 3	Advantages and disadvantages of options	
	1 About lifesaving treatment	Read
	2 About the survival rate	
	3 Ability to return to prehospital life after discharge	
	4 Mental influence of surrogate decision-makers	
	5 About the cost of medical care	
	6 About cardiopulmonary resuscitation	
Step 4	Value clarification exercise	Check and write
Step 5	Guidance in decision-making and decision	Check

### Data collection and outcome measurements

Patients were considered candidates if they decided to undergo surgery. After they completed the outpatient visit, the investigator provided written and oral explanations of the study. Candidates were provided a few days to decide on participation. If the patient consented to participate, they were instructed to contact the researchers directly. In addition, families of the candidate patients were provided with written and oral explanations of the study and were contacted later regarding their participation.

**Fig. 1:**
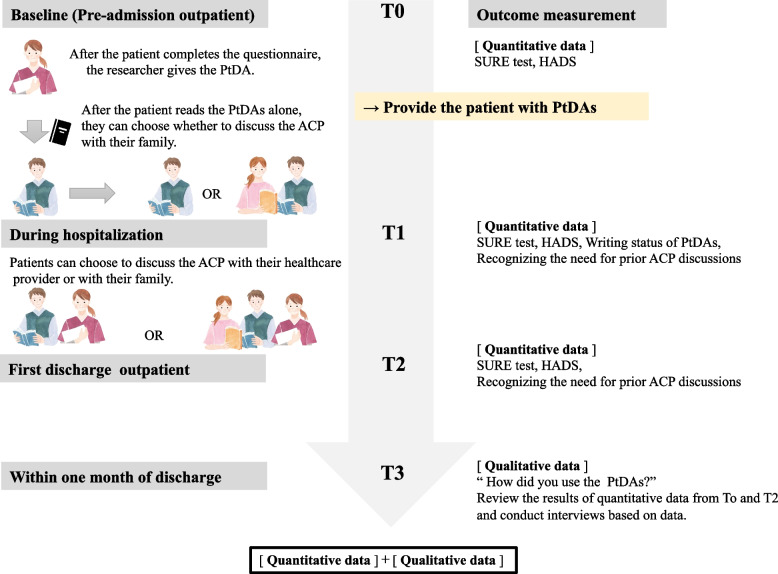
Date collection diagram. After T0, when the patient and families discussed PtDAs with a researcher, data on the conversations were collected

Data were collected from the participants at the following time points: prehospital outpatient visits (baseline_T0), during hospitalization (T1), and at the first outpatient visit after discharge (T2) (Fig. 1.). At each time point, they were asked to complete a questionnaire. Interviews were also conducted between the first outpatient visit to one month after discharge (T3). After completing the questionnaire (T0), the researchers handed the PtDAs to the patients. The researchers were not present when the patient filled out the questionnaire.

The primary outcome of this study was decisional conflict, as measured by the SURE test. The Japanese version of the SURE test [33], which assesses the quality of decision-making, was used for patient surveys [34]. This scale can assess whether a person is making a satisfactory decision based on four questions (sure of myself, understand the information, risk–benefit ratio, and encouragement) with a binary response (yes/no). Its validity and reliability have been evaluated [33, 35]. The score range is on a 0–4 point scale, with the expectation that if a patient answers ‘no’ for ≥ 1 item(s), they are likely to change their mind later and have regrets. It was a measure of whether patients use PtDAs to improve the quality of their ACP decision-making.

The secondary outcomes were as follows: perception of the need to discuss ACP before surgery, which was evaluated by a numerical rating scale (NRS; range: 0–10) and mental health status, which was assessed by the Hospital Anxiety and Depression Scale (HADS) [36]. The researchers also reviewed and assessed the options and writing status of the PtDAs. The survey administered to patients’ family members included NRS (0–10) to confirm the patient’s preferred treatment options and their perception of the need to discuss ACP with the patient before surgery and HADS to evaluate mental health status. The degree of confidence as a surrogate decision-maker was assessed by NRS (0–10). Moreover, the patients were interviewed after discharge (T3) to ascertain how they used the PtDAs pre- and post-discharge. This process was conducted to compare and assess the results of the SURE test, which is a quantitative data, with how PtDAs are used and whether they could be a tool for supporting ACP. A single researcher conducted all the interviews in a semi-structured format based on the questionnaire responses. The time of interviews ranged from 30 min to 1 h. Data on age, sex, household structure, medical history, employment, and decision-making preferences were also collected by the survey (Additional file 3).

To assess the feasibility, the following three criteria are required. (1) PtDAs should be read by patients before surgery and should be used as a tool for the investigation of ACP; (2) the timing of patient and family involvement to support ACP should be assessed and the research process confirmed; (3) the outcome of ACP support for high-risk preoperative patients should be investigated.

In addition, the extent of study participant dropout, the patient's family's participation in the study, and adverse events during the study period (Increased patient anxiety, discontinuation of surgery) were also assessed.

### Intervention using patient decision aids

Only one researcher implemented PtDAs and was involved with the patient and family at the study site, as a researcher and not as a healthcare provider. We explained to the participants that the researcher has a nursing qualification but was an external professional. During the study, researchers dealt with patients and family members in a neutral manner. The investigator and patient were scheduled to have four face-to-face meetings at the following time points: pre-admission outpatient (baseline), on admission (T1), at first outpatient discharge (T2), and an interview within one month of discharge (T3).

PtDAs were not provided until the baseline questionnaire was completed. After the baseline (T0) study, two PtDAs (PtDA_A and PtDA_B) were provided, and patients were asked to read both. Some pages of the PtDAs instructed patients to check and write as they read, following the instructions on the page as far as they could understand. If patients’ anxiety increased or if they experienced pain during the session, reading of the PtDAs was halted. The patients were informed that if they had questions, especially about PtDAs, they could always contact the study investigators.

On admission (T1), the investigator visited the patient's room and provided the questionnaire. The writing status of PtDAs was checked. The researchers did not discuss the content of PtDAs; PtDAs were discussed only when the patients asked questions, consulted, or provided topics. During inpatient visits, the patients were asked whether the researcher could visit them several times during hospitalization, and the patient who permitted them to visit the patient's room, what time the researcher could visit. During hospital visits, the researchers kept the content of PtDAs unknown and only talked about PtDAs when patients asked direct questions. At the first outpatient visit and one month (T2–T3) after discharge, the investigator contacted the patient during their outpatient visit.

### Data analysis

Basic statistics were calculated from the obtained quantitative data. Quantitative data were evaluated before and after the PtDAs were provided to the patients. SPSS Statistics (version 25.0; IBM Corp., Armonk, NY, USA) was used for statistical analyses. The qualitative data, including whether the patients were provided with the PtDAs pre-operatively, if they were read and used to understand ACP before surgery, and the reason for the patient choosing an appropriate treatment option were collected during post-discharge interviews (T3). The qualitative data were analyzed using qualitative descriptions [37, 38]. In this study, quantitative data were collected first (T0–T2), and then qualitative data were collected using interviews (T3). The results were analyzed by integrating the quantitative and qualitative data of the SURE test and the process by which patients made decisions using PtDAs.

### Ethical considerations

Participants provided written informed consent. They were aware of the methods, data management, access to their medical records, and public disclosure of results. All study participants provided written informed consent. In addition, the study was cautiously conducted, with the assumption that patients and their families would be more anxious about surgery, to avoid increasing their mental burden. The Ethics Board of St. Luke’s International University approved the study protocol (approval number: 21-A007).

## Results

**Fig. 2:**
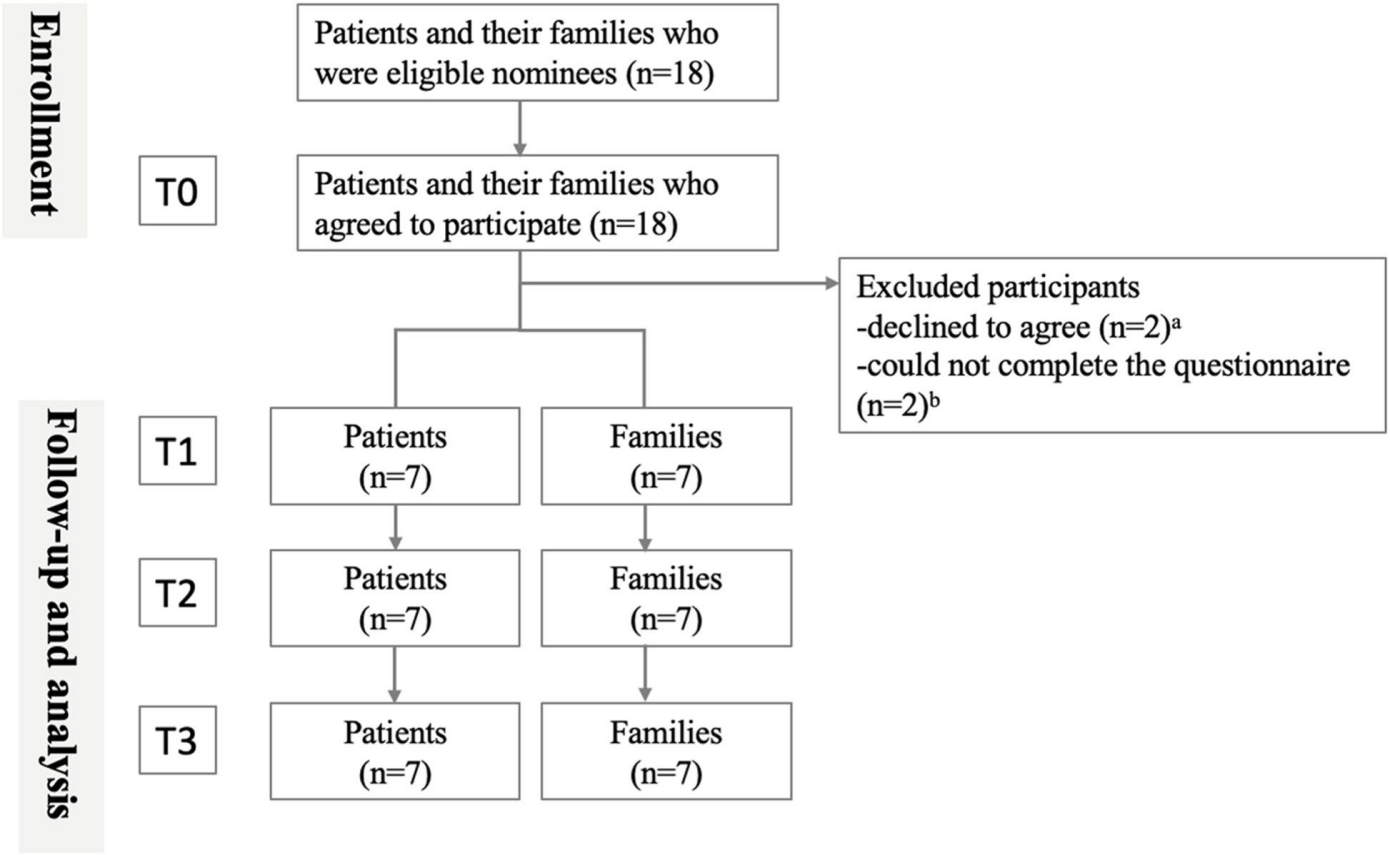
Diagram of study flow. ^a^One patient had a family member who declined to participate in the study after obtaining consent to hospitalization. ^b^One patient was excluded because although he/she could read the PtDAs, he/she could not answer the questionnaire

Seven patients and seven family members were included in the analysis (Fig. 2). Table 2 provides an overview of the patient characteristics. One patient declined to participate in the study after obtaining consent because of his family's opposition to the study. No adverse events occurred during the study due to patients stopping surgery or due to increasing anxiety.

**Table 2:** Characteristics of patients and their families dyads (*N* = 14)

Characteristics	Patients	Families
	**(*n* = 7)**	**(*n* = 7)**
Age, years, mean ± SD (range)	61.1 ± 11.6 (39–75)	49.9 ± 13.2 (30–66)
Sex		
Female	5	3
Male	2	4
Patients with cancer	6	
Stage I/II	4	
Stage III/IV	2	
Medical histories		
Yes	7	
No	0	
Employment		
Full-time	3	3
Part-time	1	1
Unemployed	3	2
Past experience with relatives’ end-of-life decision-making		
Yes	1	2
No	6	5
Relationship with patient		
Partner		5
Children		2
Time spent in meetings with the researcher, min, mean (range)		
Before hospitalization	0	0
On admission	20 (5–40)	10.7 (5–40)
During hospitalization	30.7 (15–65)	12.4 (0–30)
After discharge	52.8 (40–60)	47.1 (15–60)
Decision preference		
The physician will decide, but I will consider my opinion	5	
I will decide, but I will consider the physician's opinion	2	

The mean (± SD) age of the patients was 61 ± 11.6 years, and that of the family was 49.9 ± 13.2 years. For patients with cancer, curative surgical treatment was planned. No patient was diagnosed with postoperative complications; however, one underwent exploratory laparotomy only because the elective surgery was not performed owing to the disease progression. One patient had heard or knew about ACP at the time of the preoperative outpatient visit (T0) before PtDA implementation. The patients or their families did not ask the researcher any questions during the period between the prehospital outpatient visit (T0) and the time of admission (T1).

Seven patients responded that they had read all of PtDA_A by the time of admission (T1). Six patients read PtDA_B, and one of them read the rest with the researcher during hospitalization. All seven patients were able to check all items in the PtDA_A. Four patients filled out the free entry space. Five patients were able to check all the items in PtDA_B. Based on the answers, all the patients found it difficult to select the details of life-prolonging treatment by themselves.

Four patients used PtDAs with their families during the period from baseline (T0) to admission (T1). In addition two patients carried out discussions with their families and the researcher at admission. Furthermore, in the post-discharge survey, two patients had carried out discussions with their families and researchers, and by the time of discharge, one patient had carried out discussion with the researcher. Six of the seven patients had an opportunity to discuss ACP with three family members and investigators at discharge (T2).

Although the researchers did not propose to talk about PtDAs or ACP during their stay in the hospital, five patients started talking about PtDAs or ACP. In addition to discussing ACP, patients talked to the researchers about their course of treatment and recovery. Some patients described their needs in the perioperative healthcare support system. None of the patients scored four on the SURE test of PtDA_B at baseline (T0) (Table 3). However, on admission (T1), four patients scored a perfect score, and after discharge, six scored a perfect score.

**Table 3:** Decision-making outcomes of the study participants (*N* = 14)

	Group1		Group2		Group3		Group4		Group5		Group6		Group7	
	P	F	P	F	P	F	P	F	P	F	P	F	P	F
PtDA_A, SURE test score														
Before hospitalization	1		2		3		1		4		1		3	
On admission	4		3		4		3		4		4		2	
After discharge	4		4		3		4		4		4		4	
PtDA_B, SURE test score														
Before hospitalization	3		3		3		1		3		2		1	
On admission	4		3		4		4		4		4		3	
After discharge	4		4		3		4		4		4		4	
HADS-A score														
Before hospitalization	9	6	7	2	8	6	5	5	18	13	10	16	10	8
On admission	8	6	6	3	4	3	3	4	17	11	7	20	2	5
After discharge	7	3	1	3	4	2	2	10	13	7	7	14	8	6
HADS-D score														
Before hospitalization	5	1	1	0	8	3	5	5	13	6	4	14	4	3
On admission	3	1	1	0	3	0	5	5	12	4	3	15	1	2
After discharge	4	0	1	0	4	3	5	6	9	10	1	10	4	2
Recognizing the need for prior ACP discussions ^a^ On admission → After discharge	8 → 10	10 → 10	8 → 9	7 → 7	10 → 9	10 → 10	10 → 10	10 → 8	10 → 10	10 → 10	7 → 8	10 → 10	8 → 8	10 → 8
Confidence in proxy decision-making ^a^ Before hospitalization		9		7		7		5		9		3		3
On admission		10		7		10		10		10		6		7
After discharge		10		7		10		8		10		6		8
Patient and family discussion	with discussion		with discussion		not discussion		with discussion		with discussion		with discussion		with discussion	
Concordance of decision-making	○		○		×		○		○		○		○	

*P* patient, *F* Family, *HADS-A* HADS subscale for anxiety, *HADS-D* HADS subscale for depression

^a^ Numerical Rating scale (range: 0–10)

Patients who scored < 4 points at discharge (T2) could not read the PtDAs by themselves. Patients had the highest scores for baseline HADS-A, HADS-D, and HADS-T, and the scores decreased gradually. No patient requested to discontinue surgery because of this intervention. In contrast, the scores of the family members did not decrease according to the treatment process and increased slightly after discharge.

Patients were also interviewed after discharge about how they used the PtDA (Table 4). From baseline to admission (T1), some patients mentioned increased anxiety about the surgery. In addition to the fact that they were able to make their own decisions using PtDA, many respondents expressed higher satisfaction with being able to express their feelings to their families. Indeed, when patients and their families discussed ACP, the SURE test scores of the patients were out of 4, and there was concordance between quantitative and qualitative data.

**Table 4:** Patient’s process with patient decision aids for advance care planning

Category	Subcategory
**Before hospitalization**	
I want to think about who to use PtDAs with	Select people one can trust
	I want to think by myself first
	Patients worry about how much to tell their family, including information about one's illness
	Think about what to tell whom
	Think along with own feelings
	be unwavering in decision to operate
	I don't want to use the PtDAs alone
I want to answer for life-prolonging treatment myself	Clear one's mind
	I can't discuss about life-prolonging treatment's needs when I can't decide for myself
Worry about how to tell one's family about life-prolonging treatment	I recognize it's important to talk to my family
	I understand the need to tell my family, but I can't
	Agonize over whether to tell my family about my feelings
	Express my feelings while checking the reaction of my family
	Plan the timing and sequence of talking to my family in myself
PtDAs aid to clear my values and desires	Have your healthcare provider or family members respect my wishes?
	Expressing one's values and wishes is a first experience, and difficult
	I want to be prepared so that my family will not be in distress in case of a sudden turn over the worse
Patients have difficulty discussing with their families	Sense the concerns and anxieties of family members
	Look for a chance to have a conversation with one's family
	Care for each other
	Look for positive topics while peer round checking on one's family
	Believe that unconcealed communication leads to trust
	Look for ways to cope with one's anxiety
	Be never able to hear the true feelings of one's family
Be aware of my negative feelings	Be concerned that the use of the guide will trigger hidden fears about surgery
	Be distressed to imagine family grieving by telling them how my thinking
Relationship with healthcare providers	Start looking for a trusted healthcare provider who can express one's feelings
	Be unable to express one's uneasiness directly
	Not yet have enough trust to want to share my fears
Use PtDAs to collect new medical information to assist decision-making	Re-examine and better understand the treatment
	Share information about treatment with my family
	Understanding again that there is a risk, but don't hesitate to undergo surgery
	Take time to consider treatment options for prolonging life
**On admission**	
Express one's feelings to someone one can trust	Tell your healthcare provider that my feelings are important, but if something suddenly changes worse, I will accept the treatment my family requests
	Make it clear to the healthcare provider that one does not want life-prolonging treatment
	Express the researchers that I would like to discuss the matter with a healthcare provider again, as they may be upset if something happens suddenly worsen
Confirm one's decision	Communicate and share the decision-making process with researchers
	Communicate the discretion to the surrogate decision-maker(families)
	Relieved to have received approval from surrogate decision-makers(families), researchers / healthcare providers
**After discharge**	
Consider a relationship with health care providers	Think about who to continue discussing my ACP with
	It is considered that the same healthcare provider cannot be consulted indefinitely in an acute care hospital
	I want to be an active participant in future treatment decisions
	I want to express my thoughts on treatment to the physician
	Difficult to find myself a healthcare provider who would like to discuss ACP
Want to review the PtDAs repeatedly	Check the difference between one's decision before surgery and one's current thoughts
	I want to continue to use PtDAs to change the way I think about life-prolonging treatment when the treatment or condition changes
	I want to continue to talk to healthcare providers and my family
The satisfaction of being able to discuss my feelings with my family	Feel that one's family cares about one
	Feel accepted by one's family
	Gain the comfort of having one's feelings understood

## Discussion

To the best of our knowledge, this is the first study to investigate the implementation of ACP support in high-risk patients using PtDAs. The PtDAs used in the study were assessed for their feasibility of supporting ACP in patients scheduled for high-risk surgeries. In contrast, even if PtDAs could be provided to patients to help them make decisions, preliminary discussions between patients and their families or medical providers would be insufficient to understand and support the needs of end-of-life care patients accurately. As a research process, it was assumed that the mere provision of PtDA to patients would not prompt them to consider ACP. In addition to the usage of PtDAs, there may be a need to create a support structure that includes coaching of medical providers and opportunities for patients to review and discuss the process that led to the decision. The benefit of adding medical coaching is that knowledge about ACP can be correctly communicated to the patients and their families. In addition, the treatment that continues after surgery may encourage the patients and family members to think constantly about ACP.

In this study, PtDAs were read, written, and used by most patients prior to the admission without discomfort. It was also considered feasible because it led to the decision of the medical treatment to be received in the event of a crisis. Acceptability was also assessed because all patients and family members were highly aware of the need to investigate ACP before surgery. On the other hand, two elderly patients were excluded as they could not complete the questionnaire or PtDAs alone, and also there was opposition from family members. Before surgery, patients and family members must be provided with, understand, and submit written consent to the medical provider. However, this consent form contains a great deal of information regarding surgery. This is often burdensome for patients and has been suggested as a barrier to adequate understanding [8, 39]. Some of the study participants also said that considering ACP was stressful and that they wanted to avoid negative topics before surgery. In order to consider the burden on the patient, it is not only essential to make a decision on the medical treatment to be received in the event of a crisis, but it is also necessary to provide step-by-step support by sharing treatment goals.

The study also suggested four possible outcomes of offering PtDAs for ACP to high-risk preoperative patients. First, it may help the patients understand ACP. One outcome cited was increased knowledge of PtDAs [1]. The provision of two PtDAs to help explain the ACP process and provide knowledge about life-prolonging treatment was an opportunity to help deepen the understanding of patients and their families. ACP in high-risk preoperative patients is considered in the initiation of discussion and ACP support. ACP could be used to support early healthy stages. This study showed that ACP support for patients gave patients and their families a positive feeling that they were receiving treatment in anticipation of recovery. The patients were also satisfied with the discussion process and sharing sessions with their families and researchers. By providing the patient with an opportunity to think about ACP before surgery, the patient may be able to continue thinking about their life plan, including future treatments, and depending on the situation, discuss it with their family and health care providers to revise the treatment goals.

Second, it may help the patients to think about their values and how they want to live and receive treatment based on those desires. A previous study on ACP investigated the patient’s ability to document ADs and agreement of intent with the surrogate decision-maker [40]. The essence of an ACP is as much about the process of discussion leading to a decision, as it is about being able to make one [11, 12, 41]. The ability of patients to express their desire for treatment based on their values is essential.

Third, this provided the opportunity for the patient and the family to talk. In this study, all patients expressed their treatment thoughts to surrogate decision-makers. Only 50% of patients could initiate a discussion with their family members by themselves during the period until admission. This indicated that it is difficult for patients to initiate ACP discussions proactively. Although family members perceived discussion with patients as important, they generally avoid it [42]. The involvement of family members in ACP is essential [43], and the preferences of family members is just as important as respect for autonomy [44, 45]. Problems related to the treatment decisions for patients undergoing perioperative or critical care is because of the thin line between life-sustaining treatment and life-prolonging treatment owing to the rapid worsening of patient status and difficulties in predicting prognosis [46]. In addition, the patient’s ability to make decisions is likely to deteriorate, leading to the involvement of surrogate decision-makers and healthcare providers in the decision-making process [47]. Thus, it is important for family members, including surrogate decision-makers, to understand the patient’s treatment preferences and preferences for life-prolonging treatment. When patients want to communicate their wishes for treatment or life-prolonging treatment to family members, including surrogate decision-makers, healthcare providers should consider supporting their dialogue. This study also found that family members have as much as or higher anxiety levels than those of patients. Family members had unchanged or higher HADS scores even when patients completed the procedure successfully. Six patients had the same need for support during their treatment as their families, suggesting the importance of family care in the comprehensive implementation of ACP support for patients undergoing high-risk surgery.

Fourth, it could promote SDM. This implementation clarified that creating opportunities for patients and their families to interact with ACP themes is a challenging task for patients. An important common feature of SDM and ACP is their focus on the decision-making process and patients’ ability to decide based on their values and preferences. The use of PtDAs for ACP may facilitate SDM for ACPs by encouraging discussions between patients, family members, and healthcare providers. These four results should be considered as outcomes to assess ACP support for patients undergoing high-risk surgery and step up the study.

This study had some limitations. First, it was conducted on patients from one hospital and in one department; thus, there is a limit to the generalization of our findings to all the patients undergoing high-risk surgery. Second, our results were affected by the fifth wave of the COVID-19 pandemic, as the study was implemented when there was hospital restriction on the attendance of family members and tremendous pressure on the medical system. Third, as this study was designed to assess the feasibility of implementation by combining quantitative and qualitative data, we were unable to determine the effectiveness. The condition, thoughts, and needs of the patient who received peripheral operation and critical care, as well as their families, are subject to change. Our results indicate that it might not be able to evaluate the effectiveness as an outcome of ACP based on quantitative data alone. Future studies could improve the quality of data analysis by combining qualitative and quantitative evaluation. Here, patients undergoing high-risk surgery at acute care hospitals and their families were supported by various healthcare providers; thus, it was difficult for the same medical personnel to provide unified support to patients and their families. Therefore, in the future, it is necessary to examine the method for supporting patients and families. Our results suggested that the involvement of one investigator with patients and their families in all timelines may improve the relationship between patients and their families. For example, there is usually no opportunity for a designated healthcare provider to continue interacting with the patient during the perioperative period, and there is family other than the attending physician. Patients and their families being able to discuss ACP during the surgical treatment process may have been influenced by the ease of identification and judgment. For acute care hospitals, where it is difficult for specific nurses, other than the attending physician, to support patients and their families, better methods are needed. For example, cross-sectoral support of patient and family care by nurses who can work across organizations, such as advanced practice nurses (e.g., clinical nurse specialists), may be effective.

## Conclusions

This study suggested that ACP support with PtDAS is acceptable to high-risk preoperative patients and families and is feasible for clinical implementation. ACP support for patients undergoing high-risk surgery is important. However, it should not be the sole focus and should be comprehensively included as a necessary care in the pre- and post-discharge treatment process. These support interventions may increase patient and family satisfaction with the decision-making process. In addition to leading to acceptable decision-making as an outcome measure, initiating a patient-family discussion of ACP may also be important. To implement and disseminate PtDAs for ACP in high-risk preoperative patients, there is a need to provide support, including provision and coaching of PtDAs, and to develop procedures for supporting healthcare providers.

## Supplementary Information

**Additional file 1.** 

**Additional file 2.** 

**Additional file 3.** 

## Data Availability

The datasets used and/or analyzed during the current study are available from the corresponding author upon reasonable request.

## Abbreviations

ACP: Advance care planning
AD: Advance directive
PtDA: Patient decision aid
SDM: Sheared decision making
HADS: Hospital anxiety and depression scale
