# Supplementary material for: Implementation of advance care planning decision aids for patients undergoing high-risk surgery: a field-testing study
Source: BMC Palliat Care. 2022 Oct 12;21:179. doi: 10.1186/s12904-022-01068-2 (PMC9554854; doi:10.1186/s12904-022-01068-2)
Supplement: Supplementary file 1 — Additional file 1. [file 12904_2022_1068_MOESM1_ESM.docx]

**Additional file 1. Process of development of patient decision aids for advance care planning for patients planning to enter the intensive care unit after surgery**

**
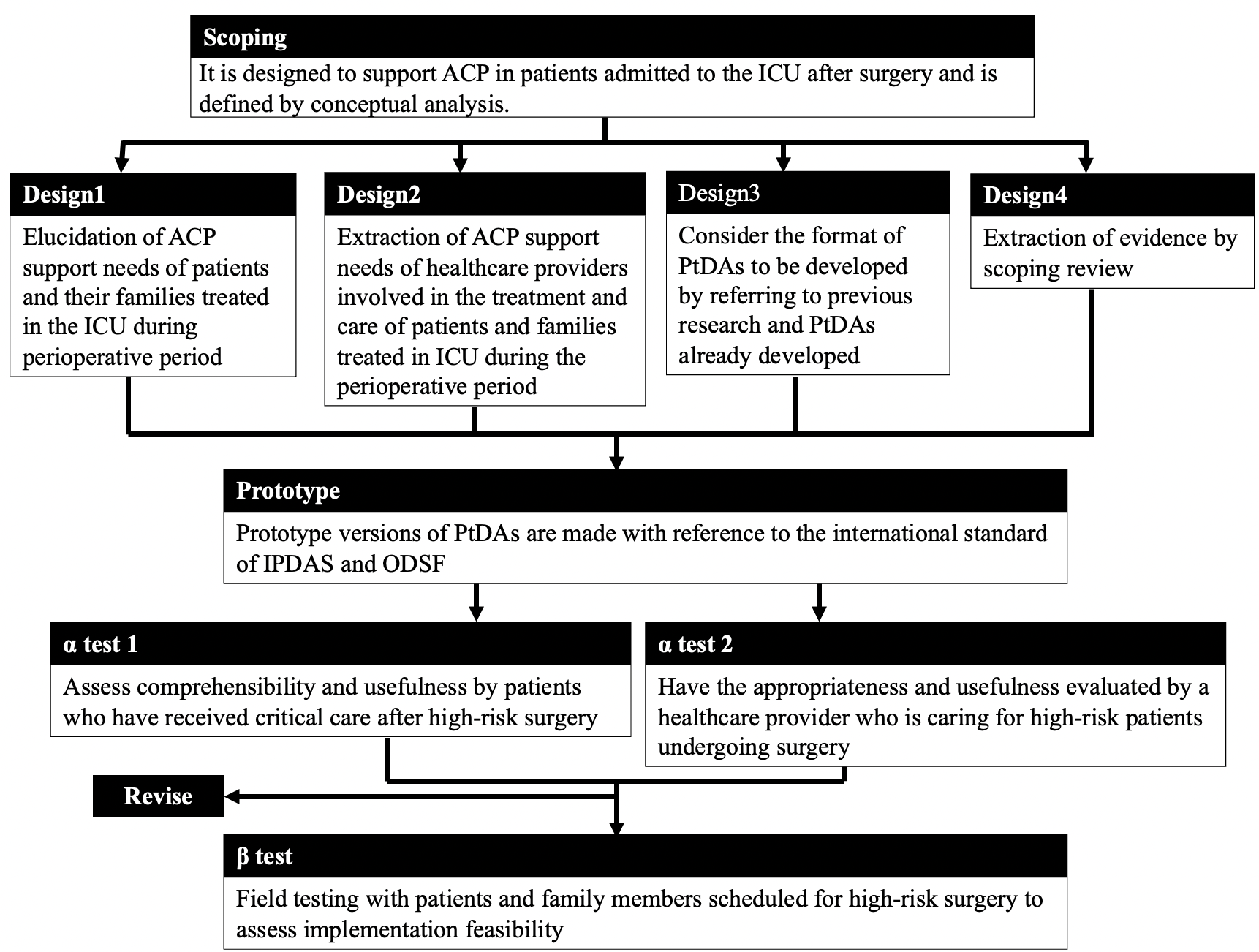
**
