# Supplementary material for: Implementation of advance care planning decision aids for patients undergoing high-risk surgery: a field-testing study
Source: BMC Palliat Care. 2022 Oct 12;21:179. doi: 10.1186/s12904-022-01068-2 (PMC9554854; doi:10.1186/s12904-022-01068-2)
Supplement: Supplementary file 2 — Additional file 2. [file 12904_2022_1068_MOESM2_ESM.pdf]

Advance care planning 1

-Think about the treatment you desire and whom you share your thoughts with -

Table of Contents

How to use the patient decision aid ... 1

STEP 1: What treatment would you like to receive?

- Whom can you share your thoughts with? ... 2

STEP 2: Think about what kind of treatment you wish to receive... 3

- About the Proxy decision-maker (trustworthy person) ... 4
- Discretionary power... 5
- What do you value and desire more? ...6

Step 3: Understand the characteristics of the options (Pros and Cons) ... 9

Step 4: Identify your priorities and decisions ... 11

STEP 5: Decide ... 13

SUPPLEMENT: Voices of Experienced People and Healthcare Professionals ... 15

How to use the patient decision aid

Structure

This patient decision aid(PtDA) consists of two books.

PtDA_A：-Think about the treatment you desire and whom you share your thoughts with -
PtDA_B：-Think about the treatment you hope for if you have difficulty recovering -

First, read Guide A while checking and writing.
STEP 5 Once you have made your decision, step up to PtDA B.

Do not worry about consulting your healthcare provider
if you feel like doing so.

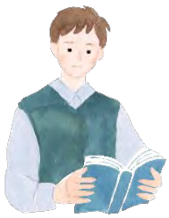

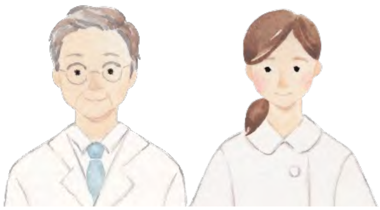

STEP 1

What treatment would you like to receive? Whom can you share your thoughts with?

Why do you not take the opportunity of surgery and treatment to make a plan for your future life and how to deal with the disease?
If you cannot decide for yourself, or if your life is in crisis, what kind of treatment would you like to receive?
This guide is designed to help you think about the treatment you want to receive in the event of a surgery.
It is also designed to help you decide whether you want to share it with your healthcare provider and the people you trust (surrogate decision-makers) .
Let us read this guide as you write.

Option

1: Do not communicate your ACP’s wishes to surrogate decision-makers and healthcare providers.
2: Communicate your ACP's wishes to surrogate decision-makers and healthcare practitioners.

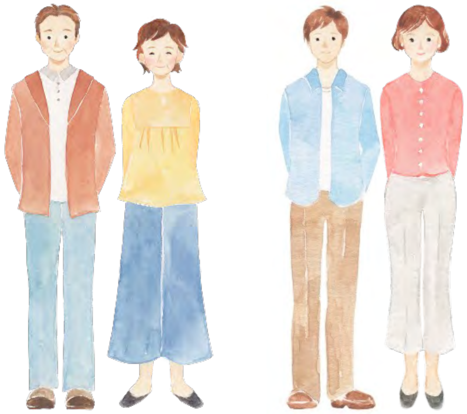

STEP 2 Advance care plannings’ process

-Think about what kind of treatment you wish to receive-

1 While you are undergoing treatment, let us think about whether you are having a hard time living.
What is important to your life?

2 Is there any treatment that you do not want to receive?
Do you think about what it would be like to die, and what it would be like to have this sort of treatment at end of life?

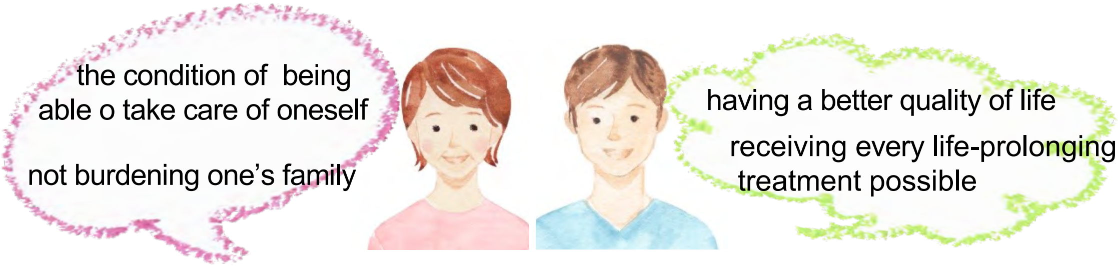

3 Next, think about whom you can trust and who can discuss the treatment and care you receive on behalf of yourself, in a crisis.
*These people are called "surrogate decision-makers."

4 You can also discuss the treatment you want or do not want with a surrogate decision-maker or healthcare provider.
This choice can be changed even after you have made a decision, depending on your situation.
If you have any questions, you can be in touch with your healthcare providers.

STEP 2

About the Proxy decision-maker (trustworthy person).

What is the role of the surrogate decision-maker (trusting person)?

When you cannot make decisions about the treatment you want rely on a healthcare provider as well as the person who is responsible for thinking and making decisions about the treatment you want to receive.

The surrogate decision-maker has no legal rights and is not involved in the division of property. Who understands your way of thinking and living the best?

In the event of an emergency, you will select a person who you believe will decide to treat you the way you want to be treated.

Spouse **Friend** **Child** **Parent**

Sibling **Healthcare provider** **Partner** **Relative**

You can have more than one surrogate decision-maker.
For example, you can choose whether you want your three children to discuss and decide, or your wife and daughter to decide.

Who is your surrogate decision-maker? Please fill it out.

Surrogate decision-maker:

STEP
2

How much do you rely on surrogate decision-makers?

What is discretion?

If you tell the surrogate decision-maker about your treatment, the decision will not always go smoothly. In particular, the surrogate decision-maker is at a loss when there is a difference between the best treatment or care for you according to the surrogate decision-maker and the healthcare provider. Therefore, it is also a good idea to decide the extent of your surrogate decision-maker’s deciding power.
For example, consider the following:

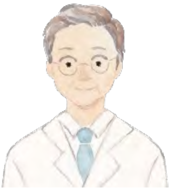

Check

What do you want to do when your decision-making ability is lost and the surrogate decision-maker or healthcare provider has a different opinion regarding the treatment?

I want you to do what I wanted. — Check ☐

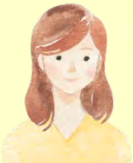

Based on the treatment I had desired, I would like the healthcare provider and the surrogate decision-maker to discuss and decide. — Check ☐

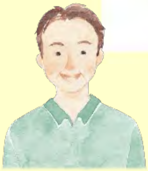

Even if the treatment is different from what I wanted, it can be decided through discussion between the healthcare provider and the surrogate decision-maker. — Check ☐

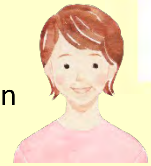

I am not sure.
I do not know. — Check ☐

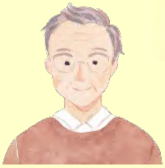

STEP 2 What do you value and desire more?

For example, let us consider the following .

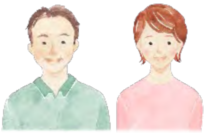

Check

If you have limited time to live, what is important to you among the following: (Multiple answers allowed)

☐ Being able to continue work and social tasks	☐ Doing what I desire
☐ Being able to take care of myself	☐ Not being a burden on my family
☐ Being financially secure	☐ Having no financial problems with my family
☐ Absence of pain or distress	☐ The state of being near family or friends
☐ Other ( )	
Reasons…	

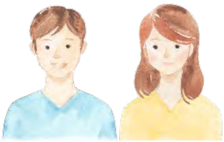

What makes you feel that it could be difficult to live in a particular situation from the following: (Multiple answers allowed)

☐ I'm in a serious condition and I cannot wake up and express my feelings to people around me.	☐ Not being able to control my body
☐ Being unable to take care of myself	☐ I cannot avoid myself
☐ Being in continuous and incurable pain	☐ Being dependent on medical equipment. (e.g., ventilator)
☐ Being unable to eat or drink by myself	☐ I do not Know
☐ Other ( )	

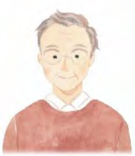

What is the predicted course of the disease if it worsens during hospitalization?

- ☐ I want to know all the information (I want to make my own decisions as much as I can)
- ☐ If it is getting worse, I do not want to know
- ☐ I do not know
- ☐ Other (   )

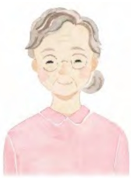

I have the following plans and pleasures that I want to pursue after I am discharged.

- ☐ Return to the job (when, timing)
- ☐ Role at home
- ☐ Travel
- ☐ Other (   )

Other than that, write down how you think and feel.

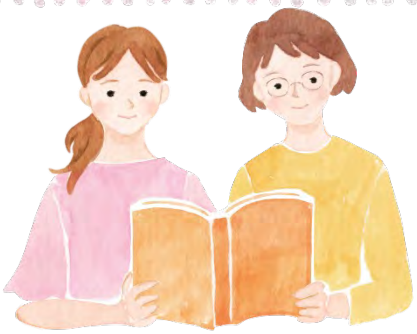

STEP 3

Understand the characteristics of the option options (Pros and Cons).

In Step 3, you will consider your options in case your life is in danger. Compare the pros and cons of discussing or not discussing your treatment with surrogate decision-makers before surgery.

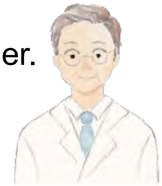

Discussion	Not discussion
Your desires are reflected in the treatment.	
8 out of 10 surrogate decision-makers fully understood the patient's desire for treatment.	1 ~ 2 of the 10 surrogate decision-makers fully understood the patient's desire for treatment.
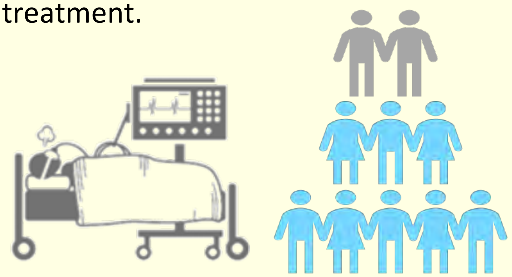	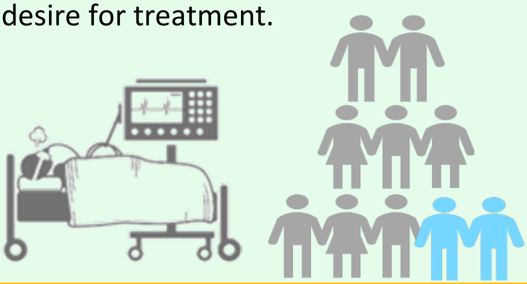
About 86% of the patients‘ autonomy was respected until the end of their lives.	About 30% of patients' autonomy was respected until the end of their lives.
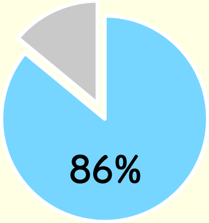	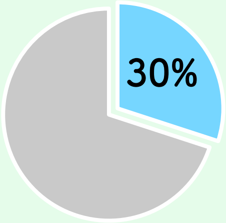

Anxiety you felt before surgery

Before surgery, you should consider how you would like to be treated in a life-threatening situation.
We compared patients who thought about it with those who didn't. Results revealed that there is no difference in the degree of anxiety in both patients.
However, in some cases, ACP before surgery may increase anxiety.

STEP 3

Understand the characteristics of the (Pros and Cons).

If you are unable to make your decision, the healthcare providers and surrogate decision-makers, who are considering treatment for you in the ICU, may experience difficulty or conflict regarding that decision.

・12.1% of conflicts within families.

・Conflicts between healthcare providers and surrogate decision-makers are reported to be 57.3%.

Discussion	Not discussion
Preoperative anxiety of surrogate decision-makers. * For the first time	
When anxiety is scored, it is reported to be 43/100. 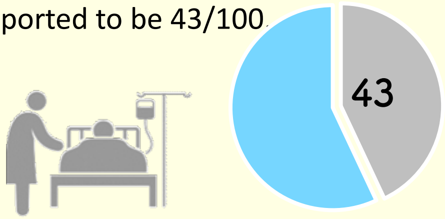	When anxiety is scored, it is reported to be 39/100. 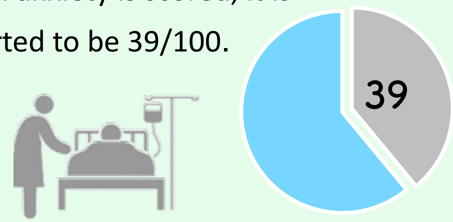
The conflict that the surrogate decision-maker has after making surrogate decisions about your treatment.	
It scored 19.5 out of 100. 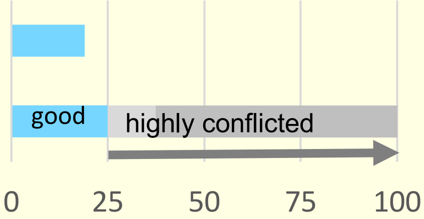	It scored 24.3 out of 100. 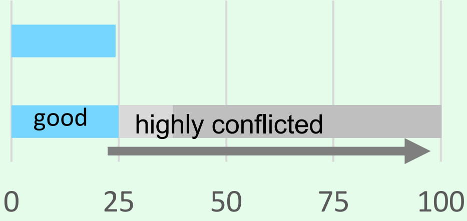
Did the surrogate decision-makers make decisions in accordance with your preferences? * Degree of confidence	
It was 8-10 out of 10. 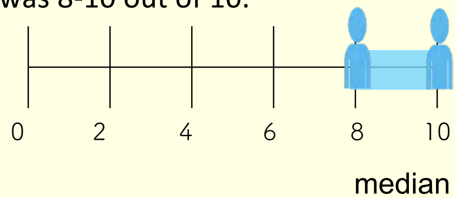	It was 5-9 out of 10. 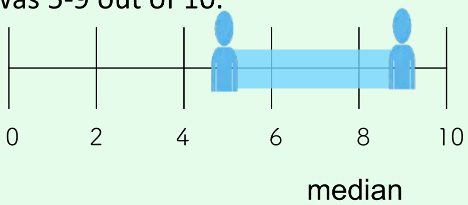

STEP 4 Examine what is most important to you

Next, you will examine what is most important to you.

For each item, check the one that best matches your preferences and see which way your value is tilted.

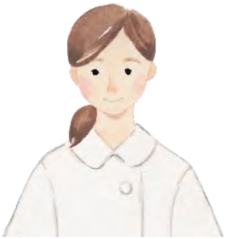

Check

Discussion	Not discussion

1. Think for yourself about the treatment you want or do not want before surgery.

☐	☐	☐	☐	☐
very important		neither		not matter at all

2. Having your surrogate decision-maker know about the treatment you want or do not want before surgery.

☐	☐	☐	☐	☐
very important		neither		not matter at all

3. Making your healthcare provider aware of the treatments you may or may not want to receive before surgery.

☐	☐	☐	☐	☐
very important		neither		not matter at all

Examine what is most important to you

Discussion	Not discussion

4. Sharing with the surrogate decision-maker information about the treatment you want or do not want before surgery may increase the surrogate decision-maker's anxiety and worry.

☐	☐	☐	☐	☐
not matter at all		neither		very important

5. That surrogate decision-makers may feel burdened by making treatment decisions on your behalf after surgery.

☐	☐	☐	☐	☐
very important		neither		not matter at all

6. When your treatment is not going well after surgery, your healthcare provider should give you any information.

☐	☐	☐	☐	☐
very important		neither		not matter at all

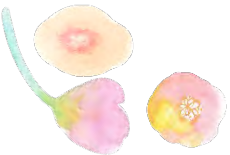

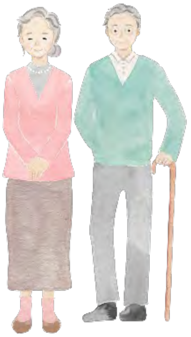

STEP 5

Decision

In the previous pages, you have considered what you value and would like to decide.
Now let us check how ready you are to decide.

Check

Do you know the benefits and risks of each option?

☐ Yes ☐ No

Do you know the benefits and risks of each option?

☐ Yes ☐ No

Are you clear about which benefits and risks matter most to you?

☐ Yes ☐ No

Do you have enough support and advice to make a choice?

☐ Yes ☐ No

If any one of the responses to the four items above is "no," you may not be ready to decide yet. Is there anything you want to do before you decide ?

STEP 5

Decision

Write down your decision,

Date：______________________

☐ Do not communicate your ACP’s wishes to surrogate decision-makers and healthcare providers.

☐ Communicate your ACP's wishes to surrogate decision-makers and healthcare practitioners.

SUPPLEMENT：

Let us take a look at the experiences of patients who have been admitted to the intensive care unit (ICU) and treated perioperative period.

Let us look at the experience of other patients. (From decision of surgery to surgery)

Let us imagine by referring to the experiences of those who have been admitted to the ICU after surgery.

Patients who never thought that their lives could have been in danger

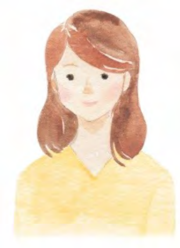

The date of the surgery was decided, so I didn't have much time before I was hospitalized. I had a young child, so I prepared so that my family wouldn't be in trouble while I was in the hospital.

It was my 2nd surgery, so I didn't feel nervous.
After I was admitted and discharged from the hospital, I worked on my schedule and plans until I returned to work, and mainly adjusted my work accordingly.
I was sorting out what I could and couldn't do while I was in the hospital.

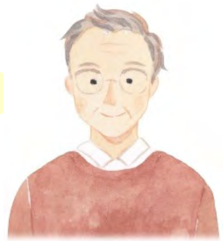

Patients who thought they could face a life crisis

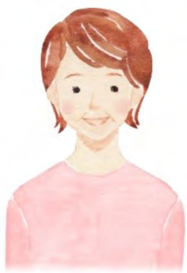

I thought I might die, so I expressed my feelings to my family. If I was going to be bedridden or I was going to be a vegetative man, let me die.
It's more like, I told them what I thought rather than discussing it.

My physician informed me that there was a high risk of complications after surgery, so I thought about my inheritance and insurance first.
I hoped that if bad happens to me, my family would not be in any trouble.

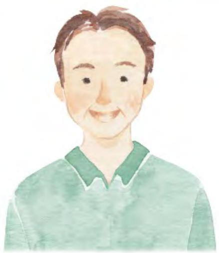

What did patients consider and express about the crisis of their lives?

Patients who discussed with one's family and loved ones

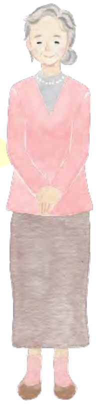

I had been informed by my physicians that there was a high risk of surgical complications, so my family was not supportive of the surgery. However, it is my life. I expressed my intention to my family that I decided to take risks. And I told my family that I asked them if there was an accident. So, on the opposite, I prepared myself for surgery.

Patients whose family was anxious and found it difficult to discuss with them

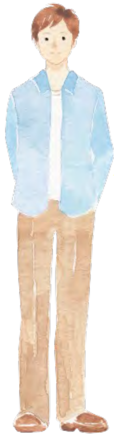

When I informed my wife of the disease, she was already crying. I couldn't talk to my wife about the future or the prospect of her death. So, I wrote a farewell note.

Afterward, I told my wife to look at it if any problems arise.

Patients who lived alone and thought they had no one to discuss with

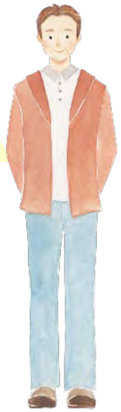

I have only one daughter. I told my daughter not to do life-prolonging treatment.

In fact, my heartbeat stopped and I almost died. At that time, I was unconscious and I don't remember anything, but my daughter and the physician seemed to think of a treatment together based on my hope for treatment.

What do healthcare providers want to know?
What do you think?

Healthcare providers have different thoughts and opinions

No treatment provides a 100% benefit. Knowing the patient's values and treatment preferences in advance will be important in the treatment plan.

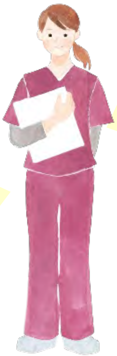

It is difficult to say to a patient who is undergoing surgery positively, "What if your life is in danger?" I think I make patients nervous. As a healthcare provider, I have the responsibility to save patients at all times.

I want the patient to recover well and be discharged. But I can't say it's 100% good. At the very least, when patients can no longer make their decisions, we want to provide them with the treatment and care they desire.

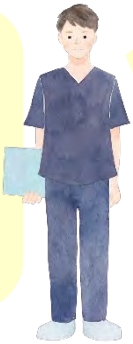

I expect the patient to be very anxious when they are informed and decide on the surgery. When I discuss ACP, I think my anxiety and worry become more intense.
I don't want to discuss it because I'm worried about the mental health of patients and their families.

Information the healthcare provider wants to know

Are there any daily activities that you value (e.g., eating by yourself or not being bedridden)?

Is there any event you want to attend? (Weddings, entrance ceremonies for grandchildren, etc.)

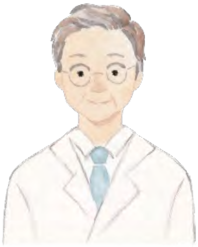

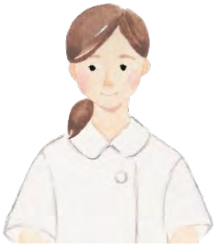

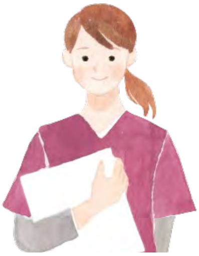

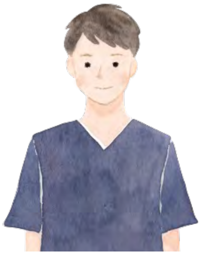

What are your wishes and pleasure in life from now on?

Who is your surrogate decision-maker? Is there any treatment you do not want to receive?

Life after discharge

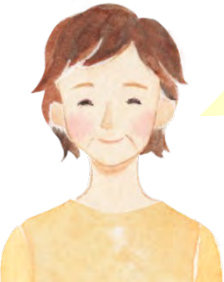

I thought about the pace of going to work as my physical condition recovered.
It is encouraging that I am getting better slowly and returning to my society. I'm doing my best, saying, “I'm at ○% return to the community.”
Words from my friends also encourage me.

I was also worried when my physician informed me that I might die or that there was a high probability of such complications.
But now, I can speak like this and do what I want to do.
I'm happy to be alive.

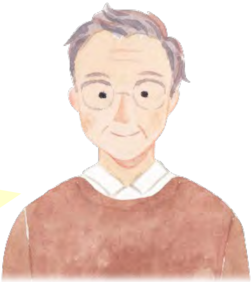

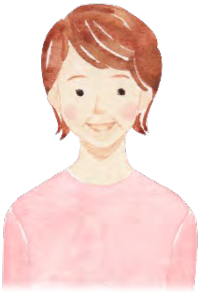

I had cancer, so the treatment continued after the surgery. I was treated with anti-cancer drugs and had two surgeries, and I'm still doing well. After the first surgery, I didn't feel sick and was able to return to work right away. What a blessing.

I returned to my job about a week after I was discharged. However, my body and mind were not ready. It didn't work at all because I took time off from work. What I can say now is that it takes more time to return to the daily life before the surgery after I leave the hospital than the surgery . It means that it was harder than the surgery after discharges' life.

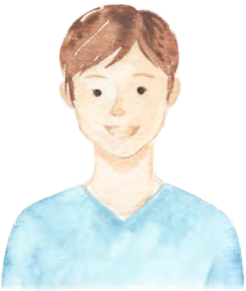

Advice from surgery after discharges' life

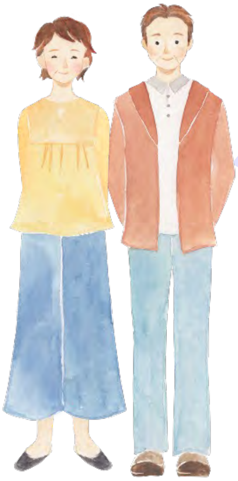

Prior to the surgery, I seriously considered my life. I think I was able to undergo a high-risk surgery because I thought about what I value and desire. I discussed with my physician until I was satisfied, and then I discussed with my family, and finally I decided by myself. I think that's important.

I think people have individual choices. I'm the type of person who chooses based on my intuition. Because I believed in my decision, I trusted myself and my physician, and entrusted my life. I think the most important thing is to be able to convince myself that it is all right.

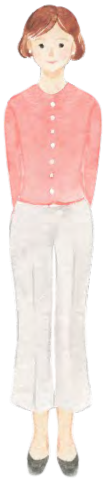

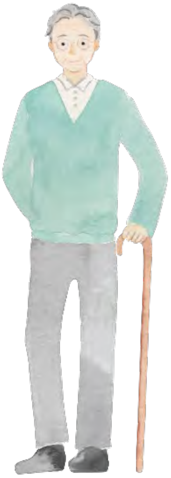

I couldn't understand the detailed explanation of the surgery. Nor could I choose what I wanted to do. It was just that I trusted the healthcare providers. I think some people get anxious when they think about various things. I think you should value what you can believe.

References

1　Health, Labour and Welfare Ministry（2018）.Medical care in the final stage of one's desired life. https://www.mhlw.go.jp/stf/seisakunitsuite/bunya/kenkou_iryou/iryou/saisyu_iryou/index.html

2　Kizawa Y. (2017). Project to improve medical systems in the final stages of life. "Advanced Care Planning: Discussion on future treatment and care”

3　Song, M.K. et al.(2005). A randomized controlled trial to improve advance care planning among patients undergoing cardiac surgery. Med Care, 43(10), 1049-53.

4　Chiarchiaro, J., et al.(2015). Perior Advance Care Planning Is Associated with Less Decisional Conflict among Surrogates for Critically Ill Patients. Ann Am thorac, 12(10), 1528-33.

5　Willson, M.K., et al.(2015). A Video to improve patient and surrogate understanding of cardiopulmonary resuscitation choices in the ICU: a randomized controlled trial. Crit Care Med, 43(3), 621-9.

6　Andreu, P., et al.(2018). Impact of a stay in the intensive care unit on the preparation of Advance Directives: Descriptive, exploratory, qualitative study. Anaeth Crit Care Pain Med.37(2), 113-9.

7　Carmen. H.M., et al(2014). Efficacy of Advance Care Planning: A Systematic Review and Meta-Analysis. JAMDA, 15, 477-489.

8　Studdert, D.M. et al.(2003). Conflict in the care of patients with prolonged stay in the ICU: types, sources, and predictors. Intensive Care Med. 29(9).1489-97.

9. Nakayama K & Iwamoto T.（2012）. Patient-centered decision support -care to make a convincing decision-

Develop by　Kanako YAMAMOTO
（St. Luke’s International University, RN, CNS, Ph.D.）
Illustrator: taneko

Created：July 10, 2020
Revised：March 10, 2021 (This PtDA is periodically reviewed and revised.)
This PtDA is supported by a JSPS 19K19613.

©2021, K Yamamoto.

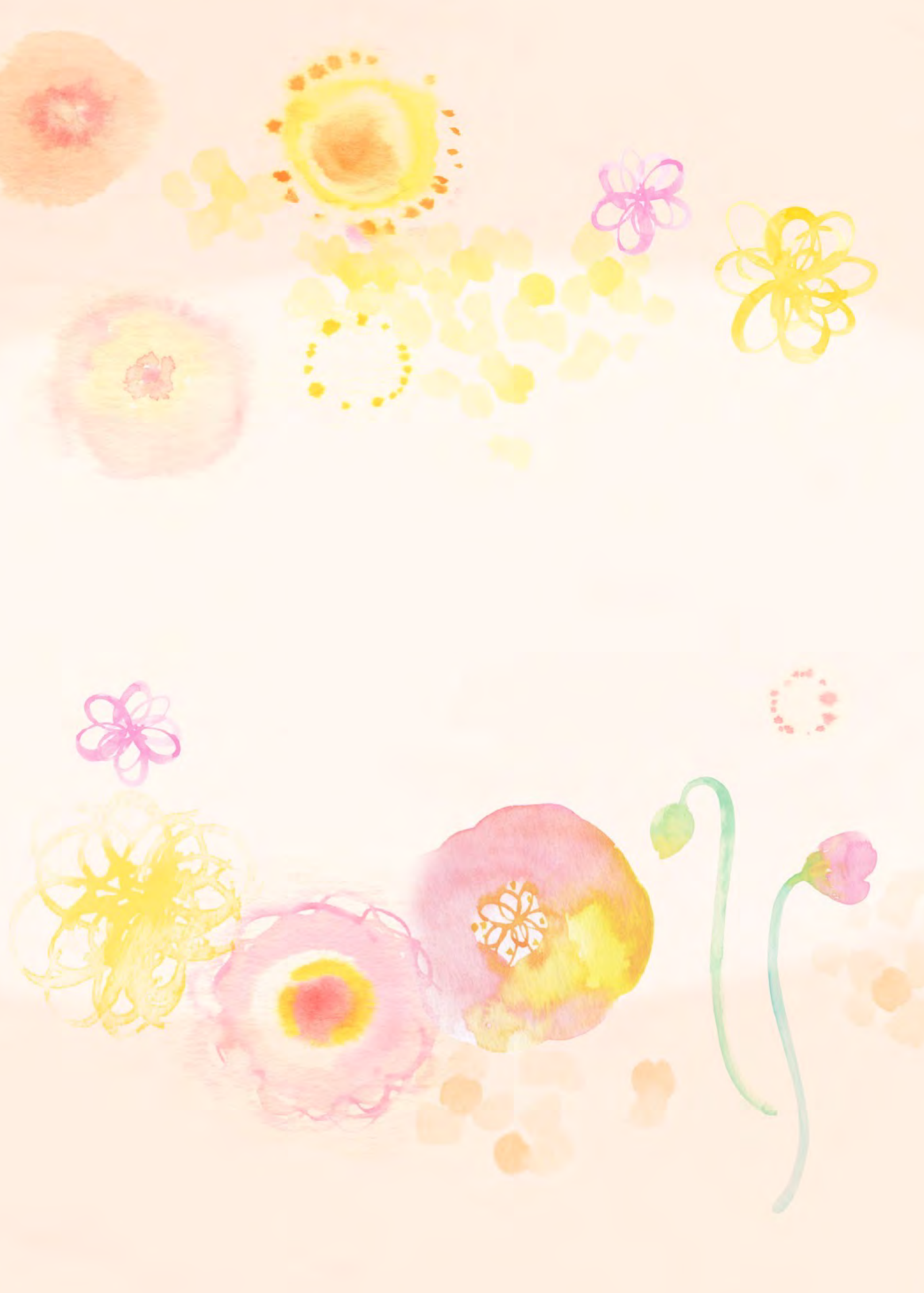

あなたが受けたい治療を考え、誰に伝えるかを

考えるためのサポートガイド

もくじ

ガイドの使い方…1

STEP 1 ：どんな治療を受けたいと考えるか、あなたの思いを誰に伝えるのか？…2

STEP 2 ：どんな治療を受けたいかを考える…3

代理意思決定者（信頼する人）について…4

あなたの代わりに治療を決める人にどの程度任せるか…5

あなたが大事にしていることは何でしょうか…6

STEP 3 ：選択肢の特徴（メリットとデメリット）を知る…9

STEP 4 ：何を大事にして決めたいか明確にする…11

STEP 5 ：決める…13

SUPPLEMENT：経験者の声・医療者の声…15

ガイドの使い方

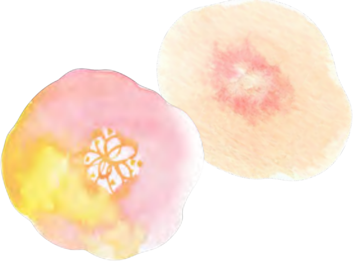

このガイドは

①「あなたが受けたい治療を考え、誰に伝えるかを考えるための
サポートガイド」と

②「回復が難しくなった場合に、どのような治療を受けたいかを
考えるためのガイド」の2冊構成になっています.

まず①のこのガイドについて、チェックをつけたり書き込みながら
読み進めてください.

STEP 5 の決めるの決定ができたら、次は②のガイドに進みます.

途中で相談したくなったら遠慮なく医療者に相談しましょう.

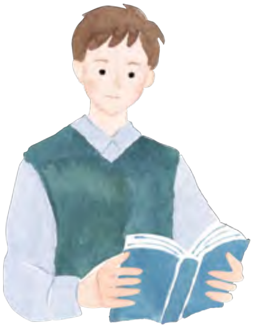

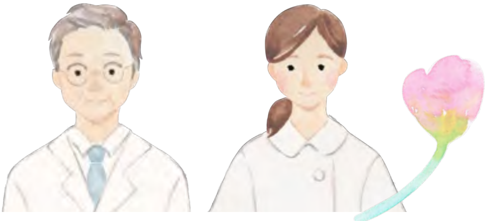

STEP 1 どんな治療を受けたいと考えるか、あなたの思いを誰に伝えるのか？

手術を受け治療を始めることをきっかけに、あなたなりの病気との付き合い方やこれからの人生計画を立ててみませんみませんか？
もしもあなたが自分で考えることができなくなったり、生命の危機的状態になった場合、どのような治療を受けたいと考えますか？

このガイドは、手術をきっかけにあなたが受けたい治療についての希望を考え、あなたの信頼する人(代理意思決定者)や医療者に伝えておくかどうかを考えるためのものです.

このガイドに書き込みながら読み進めていきましょう.

選択肢

1 あなたの思いを信頼する人（代理意思決定者）や医療者へ**伝える**

2 あなたの思いを信頼する人（代理意思決定者）や医療者へ**伝えない**

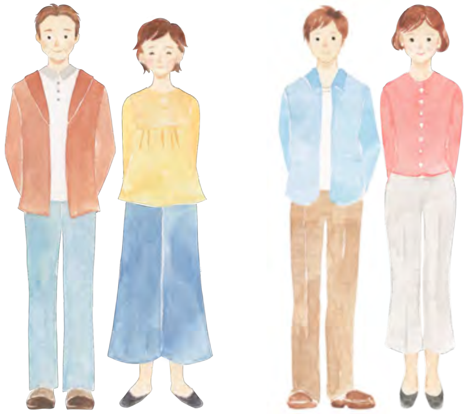

STEP 2

どんな治療を受けたいかを考える

1 あなたがこれから治療を受ける中で、もしも生きることが難しくなった場合を考え、自分の人生にとって大事な事は何かを考えてみます.

2 あなたが受けたい治療や受けたくない治療はあるかを考えます.
こんな最期だったらいいな、こんな治療や最期は嫌だな、と感じることを考えます.

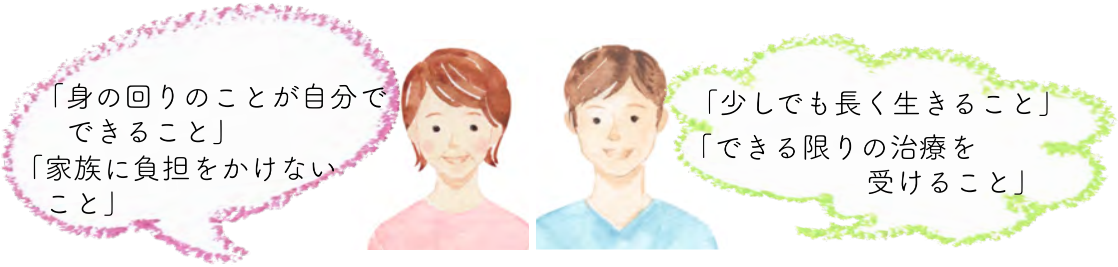

3 次にあなたが信頼することができ、いざという時にあなたの代わりにあなたが受ける治療やケアについて話し合って欲しい人は誰か考えます.
*このような人を代理意思決定者といいます.

4 あなたが受けたい治療や受けたくない治療を代理意思決定者や医療者と一緒に考えることもできます.
この意向は1度考えたら、2度と変えられないものではありません.
あなたの状況に応じて変えることができます.
また、分からないことがあれば医療者に相談できます.

STEP 2

代理意思決定者(信頼する人)について

代理意思決定者（信頼する人）とはどういう役割の人でしょうか

あなた自身が受けたい治療について意思決定できなくなった時にあなたに代わって医療者とあなたが受ける治療を考えたり決めたりする役割の人のことです.代理意思決定者には法的な権利はなく、財産分与などには関わりません.

あなたの考え方や生き方を一番理解してくれている人はどなたでしょうか.

もしもの時に、あなたがするような治療の選択をしてくれると考えられる人を選びます.

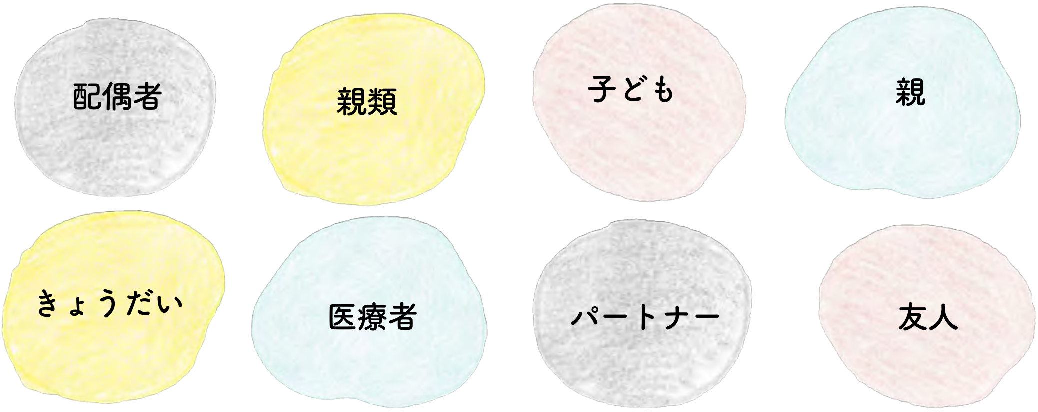

代理意思決定者は必ずしも、１人である必要はありません.

例えばお子さんが3人いらっしゃる方であれば、３人のお子さんが話し合って決めてほしい、妻と長女で決めてほしい、という選択も可能です.

<u>あなたにとって、代理意思決定者はどなたですか.記入してみましょう.</u>

代理意思決定者：

STEP 2 あなたの代わりに治療を決める人に、どの程度任せるか(裁量権)について

あなたの代わりに治療を決める人にどの程度任せるか(裁量権)とは
どのようなことでしょうか？

代理意思決定者にあなたの治療についての思いを伝えておいたとしても、必ずしもスムーズに代理意思決定が進むとは限りません．
特に、医療者(医師や看護師)が考える、あなたにとって一番良いと思われる治療やケアの内容が食い違っている時、代理意思決定者はその判断に迷います．そのため、どの程度代理意思決定者に任せるかも決めておくと良いでしょう． 例えば、以下のように考えます．

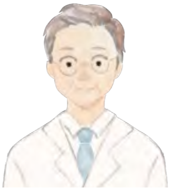

チェック

あなた自身で受けたい治療を決められなくなった時、あなたが希望していた治療と、代理意思決定者や医療者の考えが異なる時は、どのようにしてほしいですか？

私が望んでいた通りにしてほしい． チェック

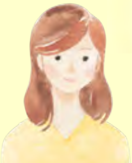

私が望んでいた治療を基本として、医療者と代理意思決定者で相談して決めてほしい． チェック

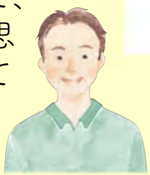

私が望んでいた治療と違っても、医療者と代理意思決定者で相談して決めて良い． チェック

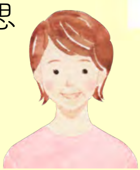

どちらともいえない．わからない． チェック

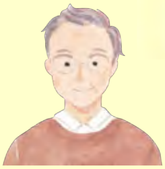

STEP 2　あなたが大事にしていることは何でしょうか

例えば、どのような内容を考えれば良いか、以下に例を挙げてみます．

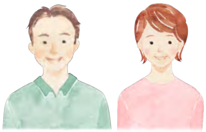

チェック

もしも、生きられる時間が限られているとしたら、私にとって大切なことは以下のようなことです．（複数回答可）

☐ 仕事や社会的な役割が続けられる	☐ 好きなことができる
☐ 身の回りのことが自分でできる	☐ 家族の負担にならない
☐ 自分が経済的に困らない	☐ 家族が経済的に困らない
☐ 痛みや苦しみがないこと	☐ 家族や友人のそばにいること
☐ その他<br>（　　　　　　　　　　　　）	
そのように考えた理由は・・・	

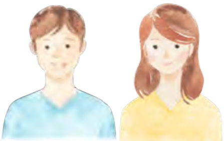

このような状態になったら「生きることが大変かもしれない」と感じるのは、以下の状況です．（複数回答可）

☐ 重体になって目が覚めず、周りの人に気持ちを伝えられない	☐ 医療機器がないと生きられない（例：人工呼吸器など）
☐ 体の自由がきかない	☐ 身の回りの事が自分でできない
☐ 自分で排泄することができない	☐ 自分で食べたり飲んだりすることができない
☐ 治すことができない辛い痛みが続く	☐ わからない
☐ その他<br>（　　　　　　　　　　　　）	

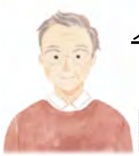

入院中に病状が悪化した場合に、予測される病気の経過は、

☐ どんな情報も全て知りたい(できる限り自分で意思決定したい)

☐ 悪化している場合、知りたくない

☐ わからない

☐ その他（　　　　　　　　　　　　　　　　　　　　　）

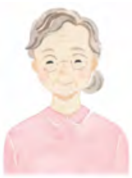

退院後の私の生活には以下のような予定、楽しみがあります。

☐ 仕事への復帰（時期：　　　　　　　　　）　☐ 家庭での役割

☐ 旅行　☐ その他（　　　　　　　　　　　　　　　　　）

その他、あなたの気持ちを整理してみましょう

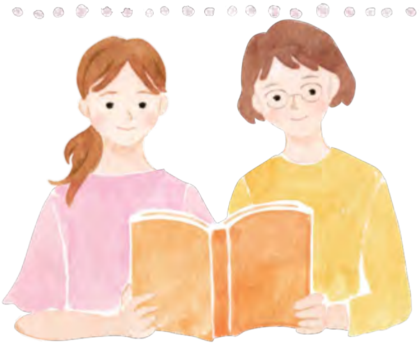

STEP 3

選択肢の特徴（メリットとデメリット）を知る

STEP3では、あなたが生命の危機的状態になった場合に備えて、手術前に代理意思決定者にあなたの治療への思いを伝えておくことのメリットとデメリットを比較して考えてみます.

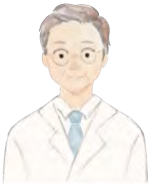

あなたの思いを伝える	あなたの思いを伝えない
あなたの思いが**治療に反映されること**	
**10人中8人**の代理意思決定者が、患者の治療への思いを完全に理解できていました. 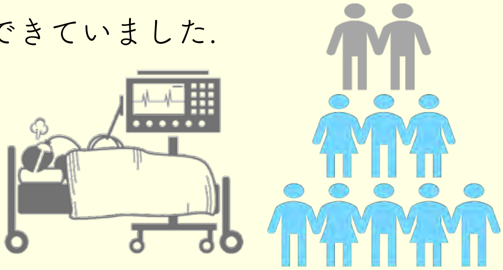	**10人中1～2人**の代理意思決定者が、患者の治療への思いを完全に理解できていました. 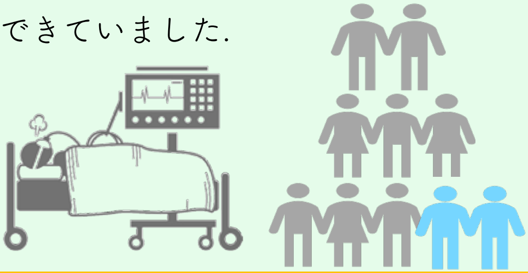
亡くなる最後まで治療への思いが尊重されていたのは**約86％**の患者です. 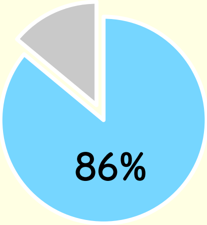	亡くなる最後まで治療への思いが尊重されていたのは**約30％**の患者です. 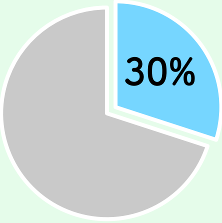
**手術前**のあなたが感じる**不安**	
生命の危機的状態になった場合にどのような治療を受けたいか、手術前に考えた患者と考えなかった患者を比較すると、**どちらの患者も不安の大きさに差はない、**という結果もありますが、**場合によっては不安が増強する**という結果もあります.	

STEP 3 選択肢の特徴（メリットとデメリット）を知る

あなた自身が治療決定ができなくなり、ICUであなたの治療決定をする時、
医療者や代理意思決定者が決定に難渋したり衝突が生じることがあります.
家族間の衝突は**12.1％**
医療者と代理意思決定者間の衝突は**57.3％**という報告があります[8)]

あなたの思いを伝える	あなたの思いを伝えない

代理意思決定者の手術前の**不安**[3)]　*初めて話し合う場合

あなたの思いを伝える

不安を点数化すると、**43/100点**であったと報告があります.

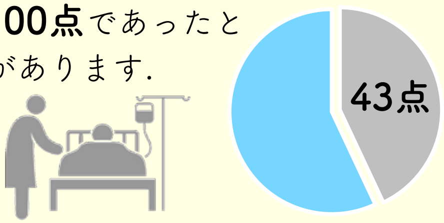

あなたの思いを伝えない

不安を点数化すると、**39/100点**であったと報告があります.

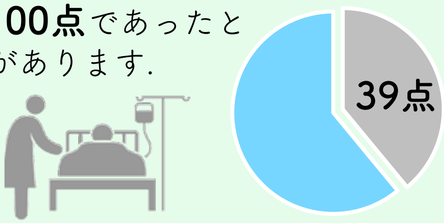

代理意思決定者が代理意思決定をした後に生じる**葛藤**

あなたの思いを伝える

100点満点中、**19.5点**でした.

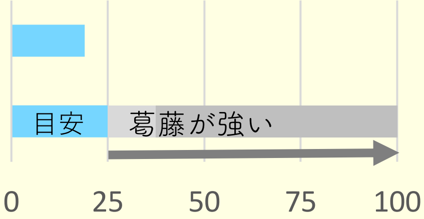

あなたの思いを伝えない

100点満点中、**24.3点**でした.

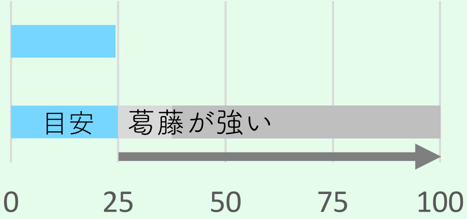

代理意思決定者があなたの意向に沿った**決定**ができたか[4)]　*自信の程度

あなたの思いを伝える

10点満点中、**8〜10点**でした.

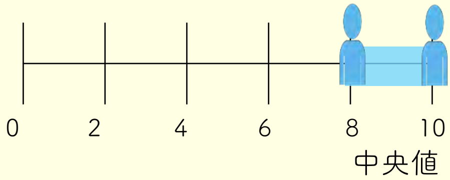

あなたの思いを伝えない

10点満点中、**5〜9点**でした.

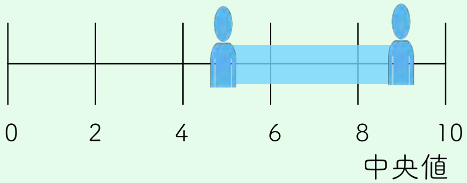

STEP 4

何を大事にして決めたいか明確にする

次にあなたにとって何が最も大切かを吟味してみましょう.
各項目について、あなたの気持ちに最も合う箇所にチェックを入れてあなたの思いを伝えるか、伝えないかどちらにあなたの価値は傾いているのか検討してみます.

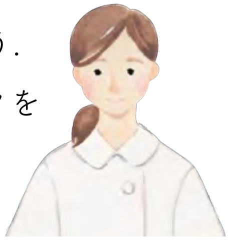

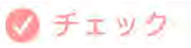

あなたの**思いを伝える**	あなたの**思いを伝えない**

1. 手術前にあなたが受けたい治療や受けたくない治療について、**自分自身で考えておく**こと

☐	☐	☐	☐	☐

とても重要　　どちらでもない　　全く重要でない

2. 手術前にあなたが受けたい治療や受けたくない治療について、**代理意思決定者に知っておいてもらう**こと

☐	☐	☐	☐	☐

とても重要　　どちらでもない　　全く重要でない

3. 手術前にあなたが受けたい治療や受けたくない治療について、**医療者に知っておいてもらう**こと

☐	☐	☐	☐	☐

とても重要　　どちらでもない　　全く重要でない

STEP 4 何を大事にして決めたいか明確にする

あなたの**思いを伝える**	あなたの**思いを伝えない**

4. 手術前にあなたが受けたい治療や受けたくない治療について、代理意思決定者と共有することにより**代理意思決定者の不安や心配が増す**かもしれないこと

☐	☐	☐	☐	☐
全く重要でない		どちらでもない		とても重要

5. 手術後に**代理意思決定者が**あなたの代わりに治療の決定を行うことによって**負担を感じる**かもしれないこと

☐	☐	☐	☐	☐
とても重要		どちらでもない		全く重要でない

6. 手術後に**治療経過が思わしくない場合、どのような情報も医療者があなたに伝えること**

☐	☐	☐	☐	☐
とても重要		どちらでもない		全く重要でない

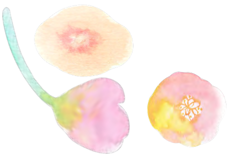

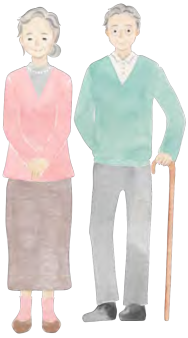

STEP 5

決める

これまでのページで、あなたにとって何を大事にして決めたいかを考えました. ここで、どのくらい**決める準備ができたか**見てみましょう.

当てはまるものに**チェック**を入れてみましょう. チェック

あなたにとって最も良い選択だという自信はありますか？

□ はい　□ いいえ

あなたはそれぞれの選択肢の利益とリスク(危険性)を知っていますか？

□ はい　□ いいえ

あなたにとってどの利益とリスク（危険性）が最も重要であるかはっきりしていますか？

□ はい　□ いいえ

この決定をするにあたって、他の人からサポートやアドバイスを十分得られていますか？

□ はい　□ いいえ

上記の4つのうち、1つでも「いいえ」がついた場合には、まだ決定の準備が十分整っていないかもしれません. 決める前にしてみたいことはありますか？

STEP 5

決める

あなたが決めたことを記入しましょう.

　　　　　　年　　　月　　　日

□ あなたの思いを信頼する人（代理意思決定者）や医療者へ**伝える**

□ あなたの思いを信頼する人（代理意思決定者）や医療者へ**伝えない**

SUPPLEMENT：経験者の声

手術を受ける経過で集中治療室へ入室し、

治療を行った経験者の声も参考にしてみましょう.

他の患者さんの経験を参考にしてみましょう（手術の決定～手術まで）

手術後にICUに入室した経験者の意見を参考にイメージしてみましょう.

自分に生命の危機的な状態が訪れるとは考えなかった人

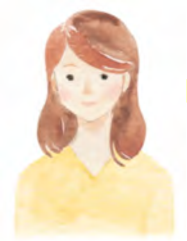

手術日が決まって、入院まで時間はありませんでした.
小さい子どもがいたので、自分が入院している間に
家族が困らないように、家の整理とか、事前に準備す
ることとかを前倒しにやったりして、家族が困らない
ように準備をしていました.

2回目の手術でしたので緊張はしませんでした.
入院して退院して、仕事復帰するまでの予定や計画
を逆算して、仕事の調整を中心に行いました.
入院中にできる仕事とできない仕事を仕分けたりも
してましたね.

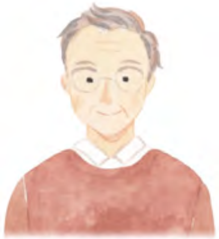

自分に生命の危機的な状態が訪れるかもしれないと考えた人

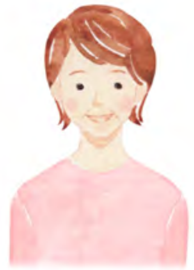

もしかしたらダメかもしれないなって頭をよぎった
ので、家族に自分の気持ちを伝えました.
もう寝たきりになるんだったら、植物人間になるん
だったらそのまま逝かせてって.話し合うっていう
より思ってることを伝えたっていう感じです.

手術後の合併症のリスクが高いってあらかじめ
主治医に言われていたので、遺産のこととか
保険のこととかをまず考えました.
自分にもしもことが起こっても、家族が困らない
ようにと思いました.

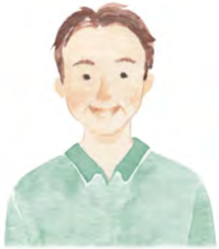

自分に「もしもの時」が起こった場合について
どのように考えて、伝えたのでしょうか

家族や大事な方と一緒に考える

合併症のリスクが高いと医師に言われており、
家族は手術に反対でした.
でも私の人生なので、リスク覚悟でやると決めたんだと、自分の気持ちを家族に伝え、もしもの時は頼んだよって話していたので逆に腹を決めて手術に臨めました.

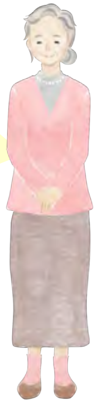

家族の不安が強く、一緒に相談するのが難しいと考えていた方

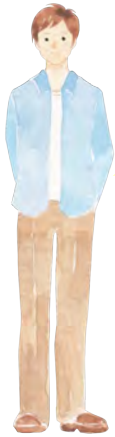

妻に病名を伝えた時点で、すでに泣いていたので、その先のことや亡くなることも想定した話はとてもできませんでした.
そのため、自分で遺書を書きました.
それで、妻にもしもの時はこれを見てねって伝えました.

現在はひとりで生活し、一緒に相談できる人がいないと考えていた方

私の身内は娘1人です。娘には、
「もしもの時は延命治療はしないでくれ」って前から伝えていました.
実際、私は心臓が止まって死にかけたことがあります. その時、私は意識がなくて全く覚えていませんが、娘と医師が私の治療の希望をもとに一緒に治療を考えてくれたようです.

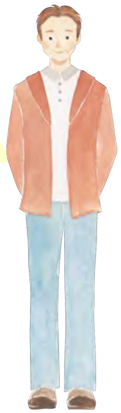

医療者は何を知りたくて、どう考えているのか？

医療者の考えや気持ちもさまざまです

100％の治療はないので、患者さんの価値観や治療の希望を前もって知ることができれば、治療計画の中で重要な情報になります．

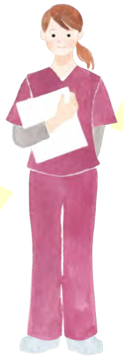

前向きに手術に臨んでいる患者さんに「万が一の時が来たらどうします？」と言うのは、不安にさせてしまうと思っています.医療者としてどんな時も絶対救命するんだっていう気持ちがあります.

患者さんに元気に社会復帰して欲しいと思っていますが、100％大丈夫とは言えないので、せめて患者さんが自分で決められなくなった時が来たら、患者さん中心の意思決定を支援したいと思います.

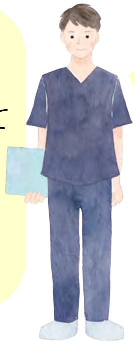

告知を受け、手術を決めた段階でも不安が強いだろうと思っています．もしもの時の話をすると不安や心配が強くなってしまうんじゃないか、患者さんやご家族の精神面が心配です.

医療者が知りたい情報

あなたが大事にしている日常生活の活動(自分でご飯が食べられる、寝たきりは嫌など）はありますか？

これからの人生の希望や楽しみが何か教えてください。
参加したいイベント（結婚式、孫の入学式など）はありますか？

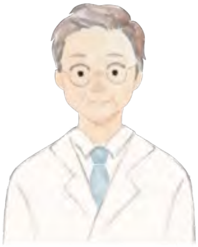

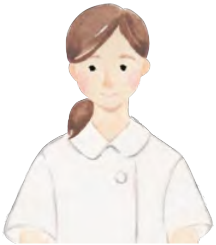

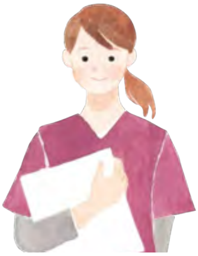

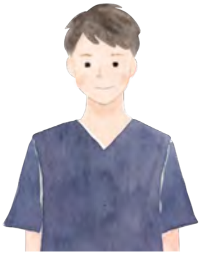

あなたにとっての代理意思決定者はどなたですか？

こんな治療だけはしてほしくない、受けたくない治療はありますか？

退院後の生活について

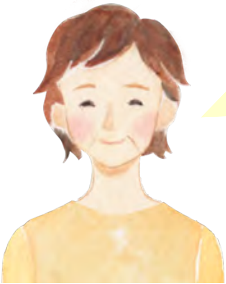

少しづつ体と相談しながら仕事に出るペースを考えました．少しずつ元気になれて、社会に戻れているっていうか、励みですよね．
自分でも、周りからも社会復帰っていう言葉を使って、「今〇％の社会復帰です」とかって言って頑張ってます．

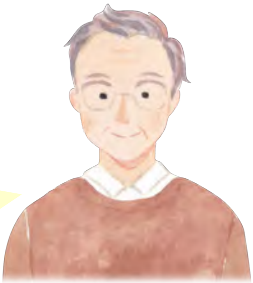

死ぬかもしれないとか、こんな合併症がこれだけ起こる可能性があるって言われて不安もあったけど、今はこうやって話もできて、やりたいことができてるって思うと、やっぱり生きていられて幸せだなって思う．

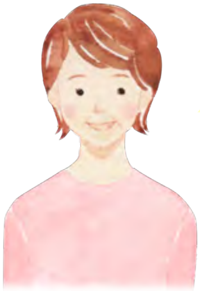

私はがんだったので、手術後の今も治療は続いています．抗がん剤をやったり、手術も2回やりましたけど、今も元気にしています．
一番最初の手術の後は、だるさもなく、すぐに仕事に復帰出来ました．ありがたいことですね．

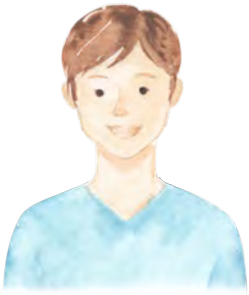

退院して1週間くらいで仕事に復帰したんですけど、身体も心もなかなかついていかなくって．休み休みで、全然ダメでしたね．
今言えるのは、手術をすることよりも、退院後に手術前の日常生活に戻るまでは時間もかかり、結構大変だったっていうことです．

先に手術を終えた人からのアドバイス

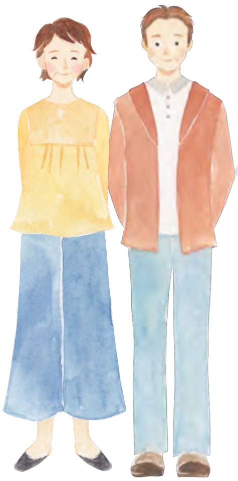

手術前は、真剣に自分のこれからの人生を考えました.
自分が大事にしてること、やりたいことを考え抜いたからこそ、手術に臨むことができたんだって思います.
主治医と納得できるまで話して、家族とも話して、それで、最後は自分で決める、私はそれが大事って思います.

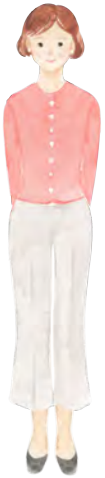

人にはそれぞれチョイスがあるって思っています.
私はピンときた直観を信じて選択するタイプで.
でも自分の決断を信じているから、自分も医師も信じて命を預けました.
自分がこれでいい、って納得できることが一番大事って思います.

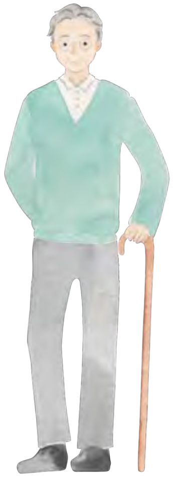

手術の細かい説明なんて、たくさん聞いてもよく分からなかったし、自分がどうしたいかなんて選べませんでした.
ただ、病院と医療者を信じる、っていうことだけでした.
あれこれ考えると不安になる人もいると思うので、自分が「信じられる」ことを大事にすればいいと思います.

引用文献

1　厚生労働省（2018）．自らが望む人生の最終段階における医療・ケア
https://www.mhlw.go.jp/stf/seisakunitsuite/bunya/kenkou_iryou/iryou/saisyu_iryou/index.html

2　木澤義之（編）平成２９年度厚生労働省委託事業　人生の最終段階における医療体制整備事業「これからの治療・ケアに関する話し合いーアドバンス・ケア・プランニングー」

3　Song, M.K. et al.(2005). A randomized controlled trial to improve advance care planning among patients undergoing cardiac surgery. Med Care, 43(10), 1049-53.

4　Chiarchiaro, J., et al.(2015). Perior Advance Care Planning Is Associated with Less Decisional Conflict among Surrogates for Critically Ill Patients. Ann Am thorac, 12(10), 1528-33.

5　Willson, M.K., et al.(2015). A Video to improve patient and surrogate understanding of cardiopulmonary resuscitation choices in the ICU: a randomized controlled trial. Crit Care Med, 43(3), 621-9.

6　Andreu, P., et al.(2018). Impact of a stay in the intensive care unit on the preparation of Advance Directives: Descriptive, exploratory, qualitative study. Anaeth Crit Care Pain Med.37(2), 113-9.

7　Carmen. H.M., et al(2014). Efficacy of Advance Care Planning: A Systematic Review and Meta-Analysis. JAMDA, 15, 477-489.

8　Studdert, D.M. et al.(2003). Conflict in the care of patients with prolonged stay in the ICU: types, sources, and predictors. Intensive Care Med. 29(9).1489-97.

参考文献

1．中山和弘, 岩本貴（編）．(2012)．患者中心の意思決定支援　納得して決めるためのケア, 中央法規

作成者　：聖路加国際大学 山本加奈子（集中ケア認定看護師, 急性・重症患者看護専門看護師）
イラスト：taneko

作成日　：2020年 7月10日（このガイドは、定期的に情報や内容を見直し改訂を行います.）
改訂日　：2021年 3月31日
2019年度　文部科学研究費補助金(若手研究:19K19613 研究代表者: 山本加奈子)の助成により作成されたものです。

©2021, K Yamamoto. <無断複写・無断転用はご遠慮ください>

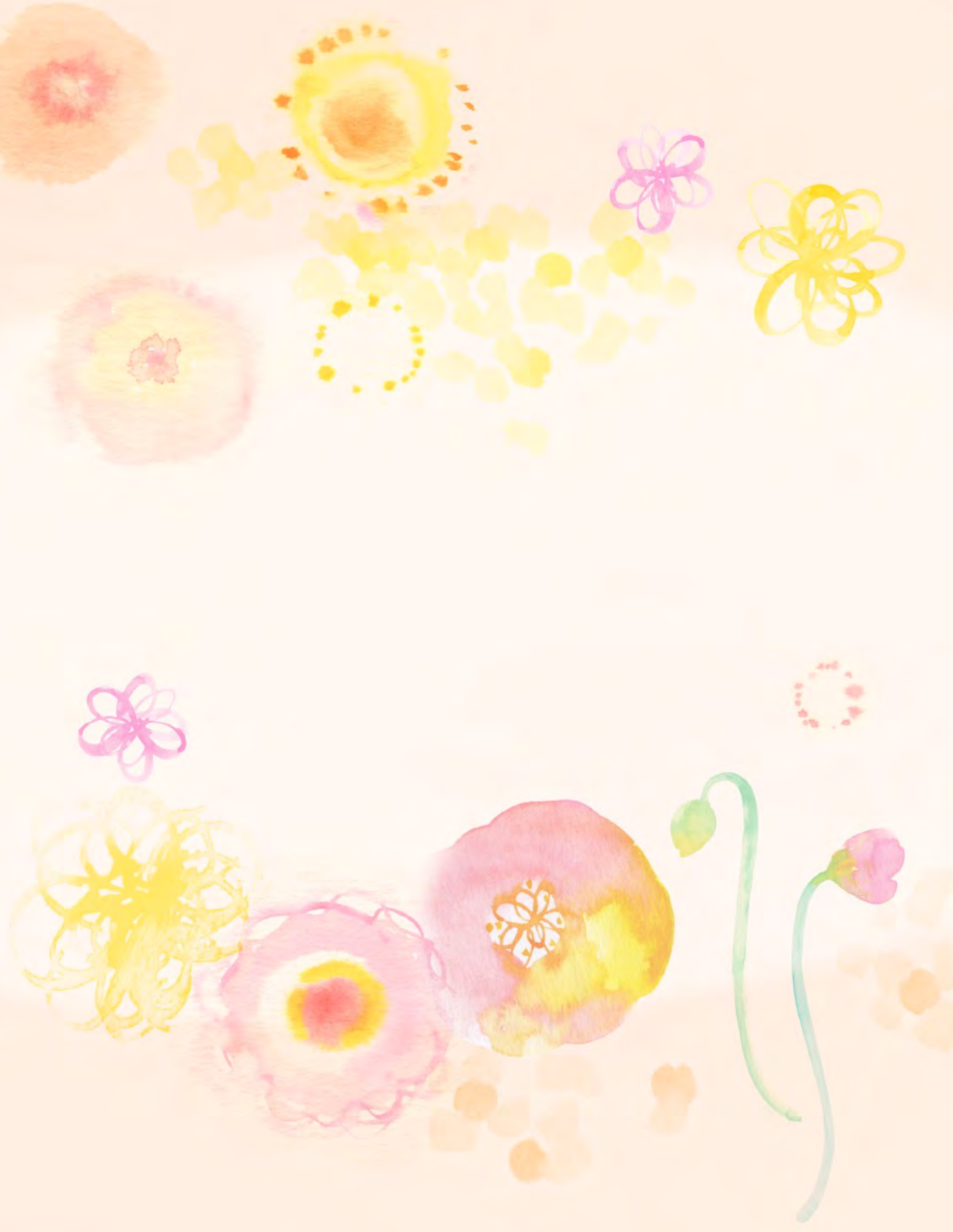

Advance care planning 2

- Think about the treatment you hope for if you have difficulty recovering -

STEP 1

Think about the treatment you hope for if you have difficulty recovering

This PtDA is designed to help you plan ahead if you are in a situation where recovery is difficult during the course of treatment and you want to make sure that you are receiving the best treatment you can until the end of your life, or if you want to limit your treatment to switching treatment goals.
As you read through the PtDA, try to clear your mind based on the options that follow.

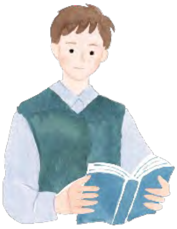

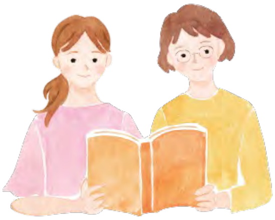

Option

1 Continue to receive all treatment regardless of the survival rate.

2 Discontinue life-sustaining treatment when the survival rate decreases.

When you are in an ICU or when your condition worsens, change happens very quickly. The time to decide treatment is very short, and it is difficult to make a careful decision. At the end of life, when recovery is considered difficult, 70% of patients are reported to be unable to make decisions on their own.

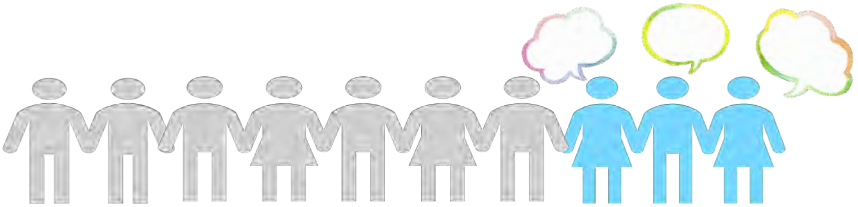

Think about your wish for treatment

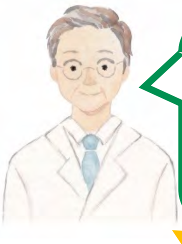

Option1
We will provide you with all kinds of treatments so that you can live as long as possible. On the other hand, you may have to be treated differently than you wish.

Option2
This means switching to treatment that focuses on relieving your pain and improving quality of life. However, this can shorten your time.

Undergo full treatment

Benefit

- You may be able to live as long as possible until your heart and breathing stop.
- Your long life will keep your family and loved ones with you for as long as possible.

Discontinue treatment that may prolong life

Benefit

- If you are unlikely to recover, you can avoid futile treatment.
- If following your wishes are no longer possible, they will be reflected and respected as much as possible.

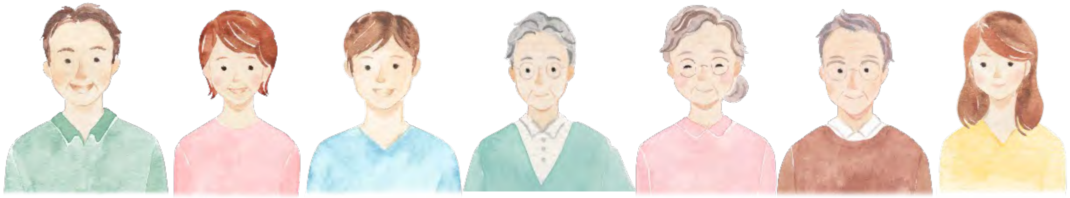

Undergo full treatment

Risk

- You cannot move freely because you are connected to a medical device. In addition, there is a high possibility that your consciousness will not recover even after treatment.
- This treatment may not be the way you wish to live.

Discontinue treatment that may prolong life

Risk

- This treatment may shorten your lifetime.
- Your family and loved ones would spend less time with you.

STEP 2 What is a physical condition with a low survival rate?

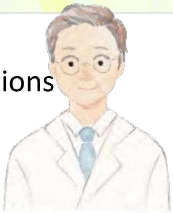

It refers to any situation where the lifesaving rate becomes low.
One case can be postoperative complications which refers to complications after surgery that can cause some of your organs to malfunction.
Furthermore, a condition in which the function of two or more organs is reduced is called multi-organ failure.

Physical condition

Brain & Nerve	If you have a stroke or bleeding, your cognitive function may deteriorate and you may not regain consciousness. The hands and feet may remain paralyzed.
Circulation	If your heart and blood vessels function poorly, you may not be able to maintain your blood pressure and pulse. This leads to a decline in the function of organs such as the kidneys, liver, and intestine.
Respiratory	There are cases where water accumulates in the lungs, pneumonia, or the lungs collapse with sputum. You cannot take in oxygen by yourself, or carbon dioxide may not be emitted, or both. In such cases, an oxygen mask or a ventilator is worn.
Liver	Poor circulation or poor liver function due to various reasons can lead to jaundice and accumulation of toxins in the body, which leads to life-threatening problems.
Kidney	Your body may hold water, and waste materials in your blood may not be able to pass out of your body. Electrolyte abnormalities can be life threatening.
Blood	If the blood fails to circulate, blood tends to clot throughout the body (clot formation), and if a wound forms, it is difficult to stop the bleeding and bleeding to death is likely.
Other	If you get infected and bacteria circulates in the bloodstream, circulation cannot be maintained. This can lead to dysfunction of various organs.
Daily life	When multiple organ damage progresses and the lifesaving rate is low, daily activities become impossible. For example, you may not be able to go to the bathroom, eat, or walk by yourself.

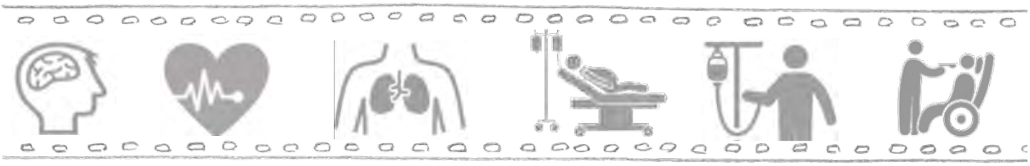

STEP 2

ICU treatment

In order for you to survive as long as possible, you may receive the following treatment in the ICU. This treatment can be brief or prolonged until your illness or symptoms improve.

Devices needed to survive. (If it is stopped, it is immediately fatal.)

Ventilator

If you are unable to breathe or inadequately breathing on your own, the machine will help you breathe by inserting a tube into your mouth or throat. *In some cases, a mouth or nose mask can be used to provide a ventilator.

Supplementary circulation device

It is applied when your heart and lung function deteriorate and you cannot maintain circulation and breathing. A tube is inserted through the groin or neck to help circulation and breathing.

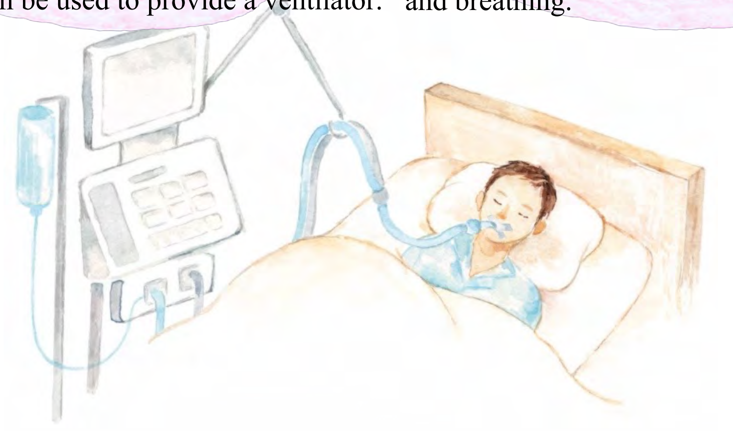

Supplementary device for living. (If you stop it, it's not immediately fatal.)

Dialysis

If kidney or liver function deteriorates, dialysis is performed at the bedside. A catheter is inserted through your groin and neck.

Sedative and analgesics

If a drain or tube is inserted, painkillers or drugs that cause sleepiness may be used.

Infusions, blood transfusion, and nutrition

You cannot drink water or eat. In that case, an infusion will be given to you. Fluid and nutrition may also be given through a tube in the nose or abdomen. You may also receive a blood transfusion.

STEP 2 What is Cardiopulmonary Resuscitation?

If the heart stops during treatment, cardiopulmonary resuscitation (CPR) is performed. This is a supplementary procedure for heart massage and artificial respiration to get the heart moving again. In general, the following occurs:

1. **Chest compression**
   We are going to press on your chest to get your heart going again.
2. **Rescue breathing**
   Open the airway, put a mask over your mouth and nose, and pump oxygen.
3. **Electric shock**
   It will send electricity to the heart so that it can move normally.
4. **Drug administration**
   Drugs to increase blood pressure are given.

*CPR usually consists of one set of chest compression and rescue breathing, not. just one or the other.

	Perform CPR	Do not perform CPR
	It is reported that about one in six patients who undergo CPR during hospitalization and can be discharged in good health regardless of disease or condition. 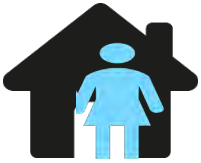	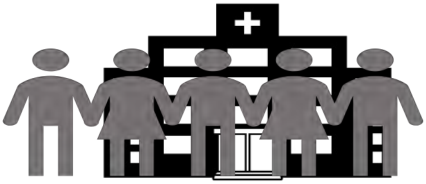
Benefit	・If CPR is successful, you can live. 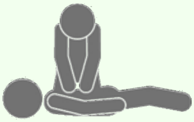	・It does not cause unnecessary injury to the body. It leads to natural death.
Risk	・If CPR doesn't succeed, you won't be able to live. The chest bones of elderly people may break by cardiac compressions.	・Without treatment, the heart and breathing do not move naturally, so you cannot live. 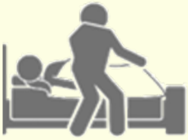

However, if the underlying disease is not treated, CPR is often not effective.

STEP 2

Discontinuation of treatment that is expected to prolong life -Focus on painless treatment

In the process of treatment, if it is judged that the survival rate is gradually decreasing and the end of life will come in a few weeks or days, the goal of treatment will be reviewed based on your wishes. Active treatment is discontinued and switched to treatment or care that focuses on relieving your pain as much as possible.

For example

Ventilator attachment and desorption repeated. Next, if breathing worsens, ventilator is not used.

Despite receiving dialysis every day, one remains unconscious. Since recovery is difficult with further continuation, dialysis is discontinued.

An assisted circulation device is installed. If the survival rate is judged to be low, the continued use of the device is discontinued.

In the ICU, patients may receive high-dose fluids and blood transfusions to maintain blood pressure. In some patients, this may cause swelling throughout the body and a significant change in the face. Skin problems may also be caused due to the insertion of a tube through the mouth for a ventilator or due to the use of many devices or tubes.

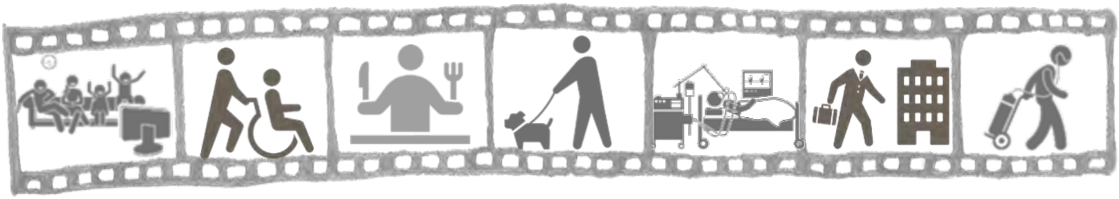

The treatment and care that you switch to are based on your medical goals and values.
Think about what living is like for you.

MEMO

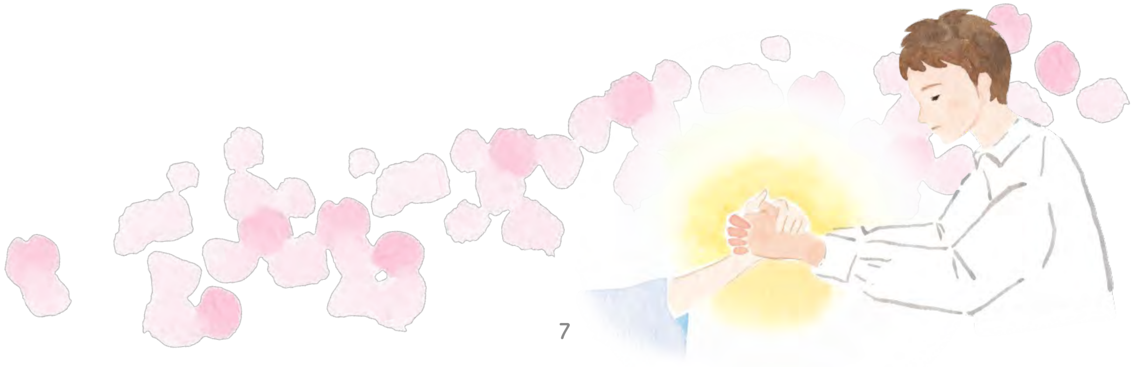

STEP 3 Understand the characteristics of the (Pros and Cons).

Let us compare treatment options when the survival rate becomes low and recovery becomes difficult during treatment.

Undergo full treatment	Discontinue treatment that may prolong life
**Content of treatment**	
It is more essential to live as long as possible, even if the treatment involves a great deal of physical and mental stress. 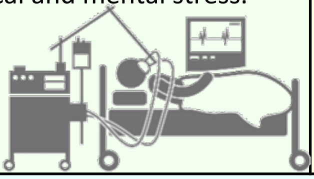	Instead of hoping that the treatment will prolong your life, it will focus on relieving pain as much as possible and treating your way of life. 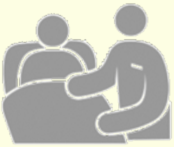
Whichever you choose, you can continue to treat your discomfort and distress with medication as long as you want.	
**Survival ratio**	
It may be possible to prolong life.<br>・The 1-month survival rate of patients with multiple organ damage is increasing. However, long-term survival (more than one year) is reported to be 37 ~ 74%.<br>Please check if you intended to use the tilde symbol (wavy dash)	・Depending on your condition, your heart and breathing may stop immediately or may stop after a while.<br>・If your survival rate is low, you may avoid unwanted treatment. Please check

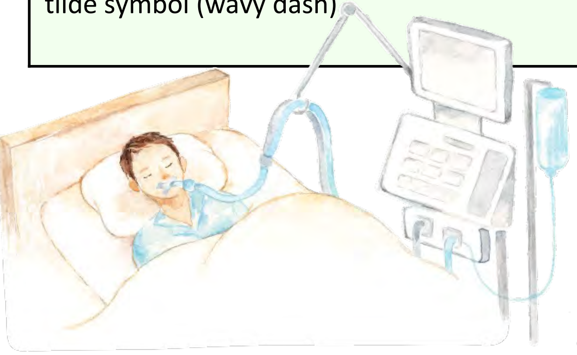

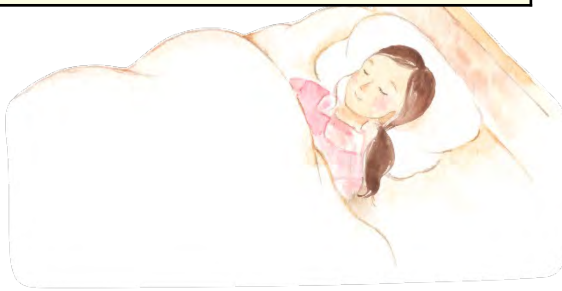

Undergo full treatment	Discontinue treatment that may prolong life
**Return to a previous life**	
If recovered, more than 70% of patients can be discharged without disability and may be able to return to previous life. 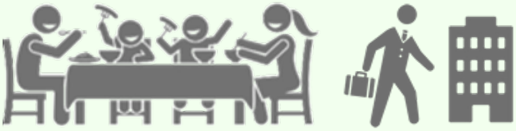	Depending on when treatment is stopped, patients may not be able to perform daily activities by themselves after discharge. 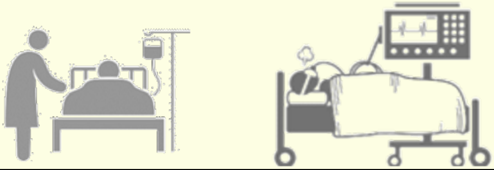
**Cognitive and mental function**	
・If recovery happens, 20-70% of patients may be discharged without cognitive decline. 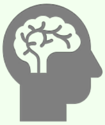 ・After treatment, 8 to 57% of patients may develop depression and anxiety. It has been reported that 50% of patients may have this symptom for a long time.	･It depends on when you stop the treatment. There is a high possibility that you cannot think and express yourself. ･The pain or discomfort may be controlled by drugs or may not be felt.

Psychological impact of surrogate decision-makers	
Between 10-80% of surrogate decision-makers may experience increased anxiety or depressive tendencies because of your admission to the ICU. 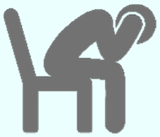	
Your recovery may reduce the anxiety and stress of the surrogate decision-maker.	When your death occurs, the anxiety and stress of the surrogate decision-maker may increase.
**Cost of medical**	
The longer you stay in the ICU or stay on life-support, the higher your medical costs.	The medical costs depend on the length of ICU treatment and the contents of treatment. 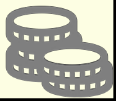

STEP 4

Clarify what you value and want to determine

Your wishes for treatment are as important as medical judgment. Let us examine what is important to you. Check the box that best matches your preference.

☑ Check

Undergo full treatment	Discontinue treatment that may prolong life

1. In this judgment, how important is your lifesaving rate?

not matter at all	☐	☐	neither ☐	☐	☐ very important

2. In this judgment, how important is it whether you can go back to your previous life?

not matter at all	☐	☐	neither ☐	☐	☐ very important

3. What is an important issue for you?

Brain & nervous	Cardiac heart	Lung	Liver	Kidney	Extremities
☐	☐	☐	☐	☐	☐
Being conscious. What you can judge, etc.	I do not want to use an assisted circulation device, etc.	I do not want to keep using a ventilator for a long time, etc.	I do not want to put on an assistive device, etc.	I do not want permanent dialysis, etc.	I do not want to be unable to walk or move by myself, etc.

Undergo full treatment	Discontinue treatment that may prolong life

4. Additional important issues or concerns for you?

☐	☐	☐	☐	☐

not matter at all　　neither　　very important

5. Are there any treatments that you really do not want to receive?

☐ No	☐ Yes

Contents：

6. Is the extent of the medical expenses an important factor in this decision?

☐	☐	☐	☐	☐

not matter at all　　neither　　very important

7. In this judgment, is it important to give priority to your opinion at all times?

☐	☐	☐	☐	☐

not matter at all　　neither　　very important

8. Is the opinion of the surrogate decision-maker important in this decision?

☐	☐	☐	☐	☐

not matter at all　　neither　　very important

9. Is the opinion of the healthcare provider important in this decision?

☐	☐	☐	☐	☐

not matter at all　　neither　　very important

STEP 5

Decision

Now let us check how ready you are to decide.

Check

Do you know the benefits and risks of each option?

☐ Yes ☐ No

Do you know the benefits and risks of each option?

☐ Yes ☐ No

Are you clear about which benefits and risks matter most to you?

☐ Yes ☐ No

Do you have enough support and advice to make a choice?

☐ Yes ☐ No

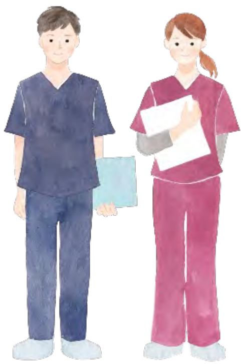

If any one of the responses to the four items above is "no," you may not be ready to decide yet. Is there anything you want to do before you decide ?

STEP 5

Decision

In the previous sections, you thought about what you would value and decide.

☐ Continue to receive all treatment regardless of the survival rate

☐ Discontinue life-sustaining treatment when the survival rate decreases.

Now let us look at how ready you are to decide.
For me, life-prolonging treatment means:

① It is the case where the lifesaving rate is considered to be about ____% according to the judgment of the healthcare practitioner.

② Others:____.

The following treatments are continued. (Check the treatment you want to receive)

☐ CPR

☐ Mechanical ventilator

☐ Drugs to maintain blood pressure

☐ Dialysis

☐ Assisted circulation device (Percutaneous cardio pulmonary support)

☐ Infusions and nutrition

☐ Blood transfusion

Write down your thoughts

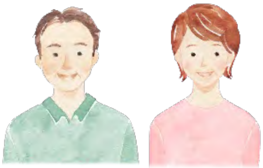

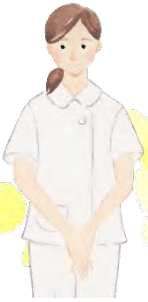

It may be difficult to decide what treatment you want to receive if recovery becomes difficult. Once you decide, there is nothing you cannot change again.
Let us do our best to get over the surgery safely.

References

1 . Advance Care Planning: Should I Stop Treatment That Prolongs My Life? (Healthwise)
[https://www.healthwise.net/ohridecisionaid/Content/StdDocument.aspx?DOCHWID=tu1430, 2020.3.4]

2 Desai SV et al. (2011). Long-term complications of critical care. Crit Care Med, 39, 371-379.

3 Hervey MA et al. (2016). Postintensive Care Syndrome: Right Care. Right Now…and Later. Crit Care Med, 44, 381-358.

4 Winter BD et al.(2010). Long-term mortality and quality of life in sepsis: A systematic review. Crit Care Med. 38, 1276-1283

5 Coulter A et al.(2013). A systematic development process for patient decision aids. BMC Med Inform Decis Mak, 13 Suppl 2:S2.

6 Nakayama K & Iwamoto T. (2012) . Patient-centered decision support -care to make a convincing decision-

Develop by Kanako YAMAMOTO
(St. Luke's International University, RN. CNS, Ph.D.)
Illustrator : taneko

Created : July 10, 2020
Revised : March 10, 2021 (This PtDA is periodically reviewed and revised.)
This PtDA is supported by a JSPS 19K19613.

©2021, K Yamamoto.

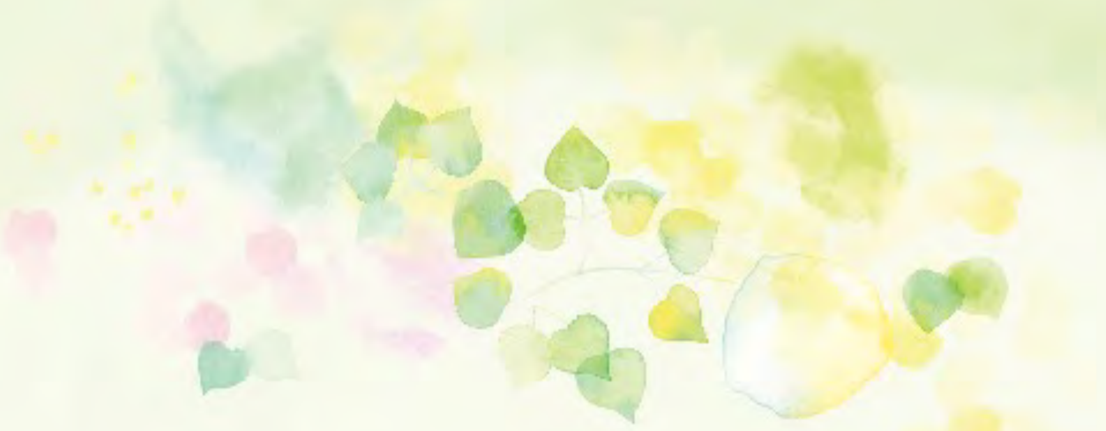

回復が難しくなった場合に、
どのような治療を受けたいかを考えるためのガイド ②

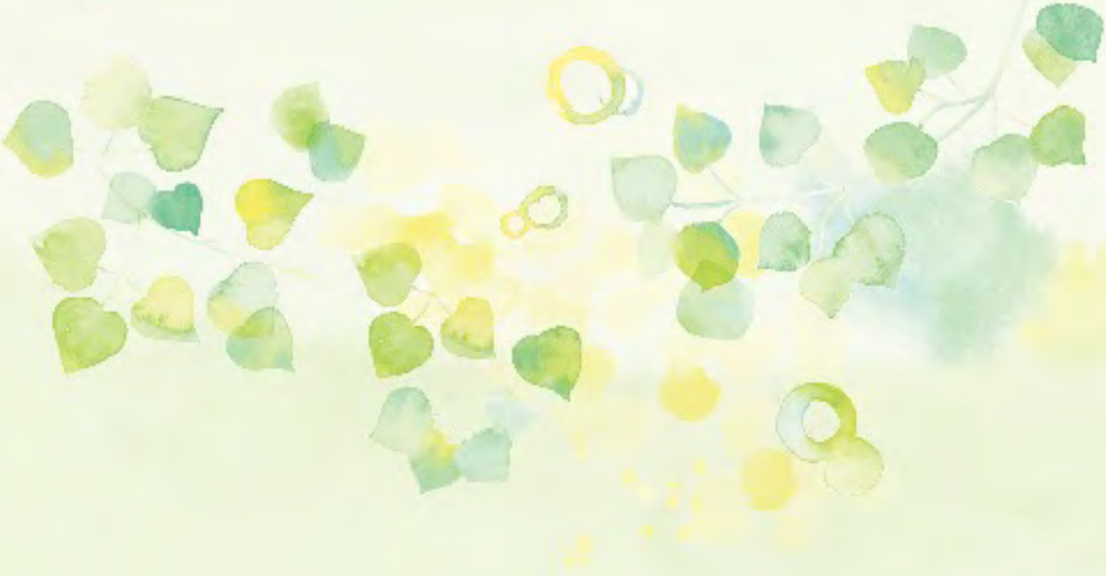

STEP 1

回復が難しくなった場合の治療を考える

　このガイドは、治療の途中で回復が難しい状態になった時に、最期までできる限りの治療を受けるか、治療のゴールを切り替え 受ける治療を制限するかを事前に考えておくためのものです.
ページを読み進めながら、以下の選択肢についてあなたの考えを整理してみましょう.

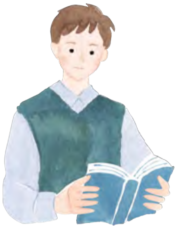

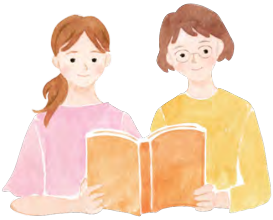

選択肢

1　救命率に関係なく、**すべての治療を受ける**

2　救命率がある程度低下したら、**延命効果を期待する治療をやめる**

　集中治療室(ICU)に入室している時や状態の悪化が起こる場合、その**変化は急激であり治療の決断までの時間が非常に短く**、じっくり**考えて決断することが難しい**特徴があります.
　回復が難しいと考えられる「終末期」の段階では、**7割**の人が自分で意思決定ができなくなると言われています[1].

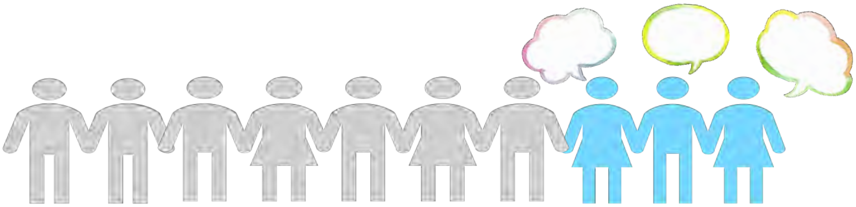

病気や治療についての希望を考えてみましょう

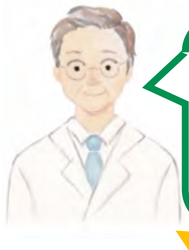

「**救命率に関係なく、すべての治療を受ける**」とは、
あなたが、1秒でも長く生きられるようにあらゆる治療を行います.
一方で、あなたの望んでいる生き方とは異なる姿で治療を
受けなければならない可能性があります.

「**救命率がある程度低下したら、延命効果を期待する治療をやめる**」とは、
あなたの苦痛の緩和や生活の質を1番重視した治療に切り替えることです.
それにより、あなたの生きる時間を短くしてしまう可能性があります.

「すべての治療を受ける」

メリット

・心臓や呼吸が止まってしまうまでは、できるだけ長く生きることができるかもしれません.

・あなたが長く生きることで、あなたの家族や大切な人があなたとできるだけ長く一緒にいることができます.

「延命効果を期待する治療をやめる」

メリット

・回復する可能性が低い場合、無益な治療をせずに済みます.

・あなたらしい生き方ができなくなった場合は、あなたの希望ができる限り治療に反映され、尊重されます.

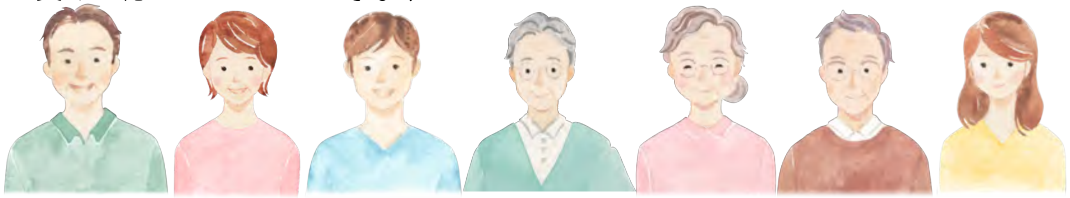

デメリット（すべての治療を受ける）

・器械に繋がれて、自由に動くことが出来なかったり、あなたの意識は戻らない可能性が高いかもしれません.

・あなたが望む、あなたらしい生き方とは異なっているかもしれません.

デメリット（延命効果を期待する治療をやめる）

・生きる時間を短くしてしまうかもしれません.

・あなたの家族や大切な人があなたと一緒に過ごす時間が短くなるかもしれません.

STEP 2

救命率が低くなる身体の状態とは

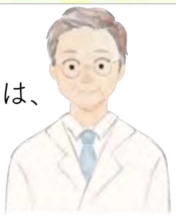

救命率が低くなる状態には、あらゆる場面が想定できますが、1例には術後合併症があります．手術の後、合併症を併発した場合には、あなたの身体の臓器のいくつかが機能不全を起こす場合があります．

2つ以上の臓器の機能が低下した状態を多臓器障害と言います．

身体の状態

脳・神経	脳梗塞や脳出血が起こると、認知機能の低下や意識が戻らないことがあります．また、手足に麻痺が残ることもあります．
循環	心臓や血管の機能低下で、血圧や脈拍を維持できなくなる場合があります．それにより、腎臓や肝臓、腸などの臓器の機能低下にもつながります．
呼吸	肺に水がたまったり、肺炎になる、痰などで肺がつぶれ自分の力で酸素を取り込めなくなったり二酸化炭素が排出できない、またはその両方の状態になることがあります．その場合、酸素マスクや人工呼吸器を装着します．
肝臓	循環が悪くなったり、別の理由で肝臓の機能が低下すると黄疸がでたり体の中に毒素がたまりやすくなり、命の危険につながります．
腎臓	体中に水が溜まりむくんだり、血液中の老廃物が体外に排出できず、電解質の異常から命の危険につながります．
血液	血液が機能不全に陥ると体中では血が固まりやすくなり（血栓ができる）、傷ができた場合、血が止まりにくく、出血しやすくなります．
その他	感染し、血液の流れで全身に菌が回ることで、循環が維持できなくなり、あらゆる臓器の機能不全につながることがあります．
日常生活	多臓器障害の進行により、救命率が低くなった状態では、自分でトイレに行ったり、食事をしたり、歩いたりすることができない状態になります．

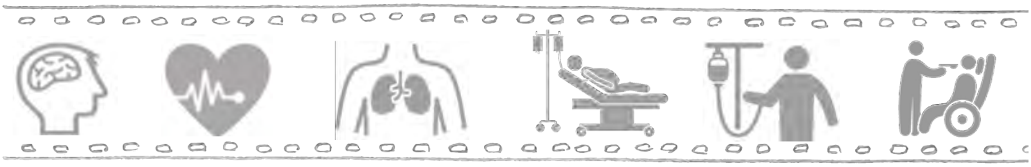

STEP 2

ICUで行われる治療

できるだけ長く生きるために、ICUで以下の治療を受けることがあります.
これらは、あなたの病気や症状が改善するまでの短時間の使用になることもあれば、長期に及ぶ可能性があります.

生きるために必要な機器（中止すると命に直結します）

「人工呼吸器」

自分の力で呼吸できない、または不十分な場合、口や喉にチューブを入れて器械が呼吸を助けます.
*場合によって、口や鼻のマスクで人工呼吸器をつけることもできます.

「補助循環装置」

心臓や肺の機能が低下し、循環や呼吸を維持できない場合は、足の付け根や首からチューブを挿入し循環や呼吸を助けます.

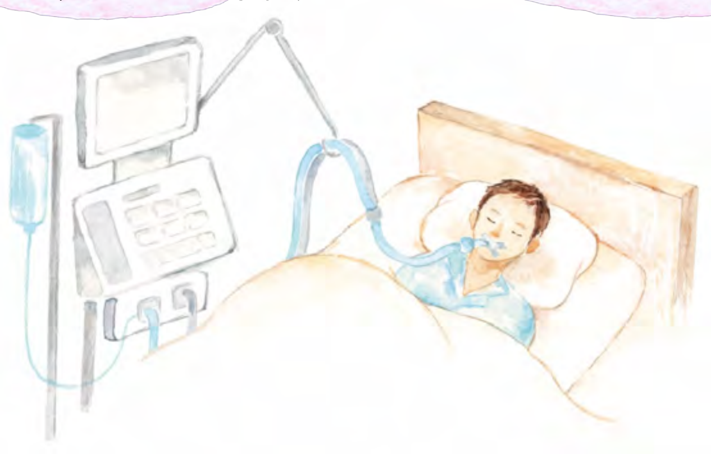

生きるための補助的な機器(中止してもすぐに命に直結しません)

「透析」

腎臓や肝臓の機能が低下した場合、ベッドサイドで透析を行います. 足の付け根や首からカテーテルを挿入します.

「鎮静剤や鎮痛剤」

ドレーンやチューブが挿入されている場合、鎮静薬（眠たくなる薬）や鎮痛薬（痛み止め）が投与されていることがあります.

「点滴や輸血、栄養」

飲水や食事摂取ができない場合は、点滴だけでなく鼻やお腹のチューブから水分と栄養を投与します.
輸血を行うこともあります.

STEP 2

心肺蘇生とは

治療中に心臓が止まってしまった場合は、**心肺蘇生**を行います.
これは、心臓マッサージや人工呼吸を行い心臓が再び動き出すための補助的な処置です.　一般的に、以下が行われます.

1．**心臓マッサージ**（胸を押し、心臓が再び動き出すようにします）
2．**人工呼吸**　（気道を確保し、口と鼻にマスクをあて酸素を送り込みます）
3．**電気ショック**　（心臓に電気を流して、心臓が正常に動くようにします）
4．**薬剤投与**　（血圧を上げるための薬を投与します）

＊通常心肺蘇生では、心臓マッサージと人工呼吸は1セットであり、
どちらかだけを行うというものではありません.

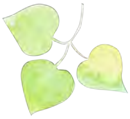

	**心肺蘇生を行う**	**心肺蘇生を行わない**
	疾患・状態を問わず、入院中に心肺蘇生を行った人のうち、元気に退院できる人の割合は**15%程度（6人に1人）**と言われています[2]. 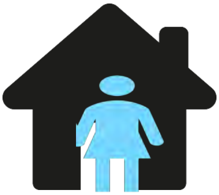	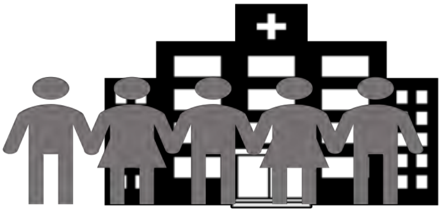
利点	心肺蘇生がうまくいくと、生きることができます. 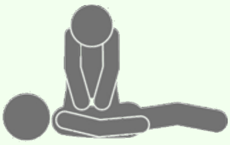	身体に不要な傷を与えることはありません. 自然な形で最期を迎えることに繋がります.
欠点	心肺蘇生がうまくいかなかった場合、生きることができません. 高齢者は心臓マッサージにより、胸の骨が折れることがあります.	処置をせず、自然に心臓や呼吸が動き出すことはまずないため、生きることができません. 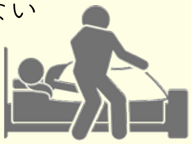

ただし、原因となる疾患が治らない場合、心肺蘇生の効果はないことが多いです.

STEP 2 延命効果を期待する治療をやめること ー苦痛がない治療を重視するー

治療を継続する過程で、少しづつ救命率が低下し人生の最期が数週間、数日で訪れるだろうと判断される場合、あなたの希望をもとに、これまでの治療の目標を見直します．積極的な治療を中断し、できるだけあなたの苦痛が少なく安楽に過ごせることを重視した治療やケアに切り替えます．

例えば・・・

人工呼吸器の装着・離脱を繰り返していたが、次に呼吸が悪くなった場合、人工呼吸器は装着しない．

連日、透析を行っていたが意識がない状態が続きこれ以上継続しても回復が難しいため、透析を中止する．

補助循環装置を装着しているが、救命率が低いと判断される場合、装置の継続的な使用をやめる．

ICUで血圧維持のために大量に輸液や輸血をすることで全身がむくみ、顔貌が大きく変化してしまう方がいます．また、人工呼吸器装着のために口から管を入れたり、たくさんの器械、チューブ類を装着することで皮膚トラブルを生じることもあります．

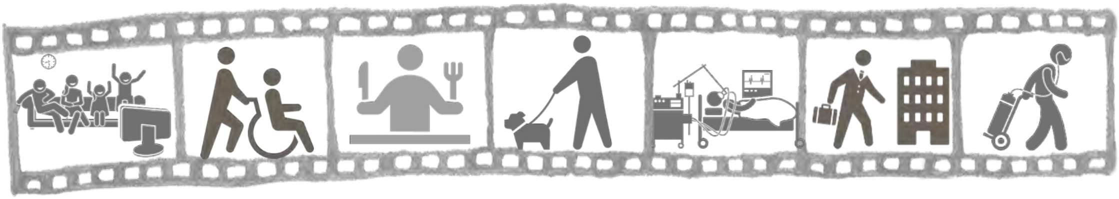

具体的にどのような治療やケアに切り替えるかは、あなたの治療の目標と価値観をもとに、医療者と考えます．
あなたにとって、「生きる」ことはどういう状態なのかを考えてみます．

MEMO

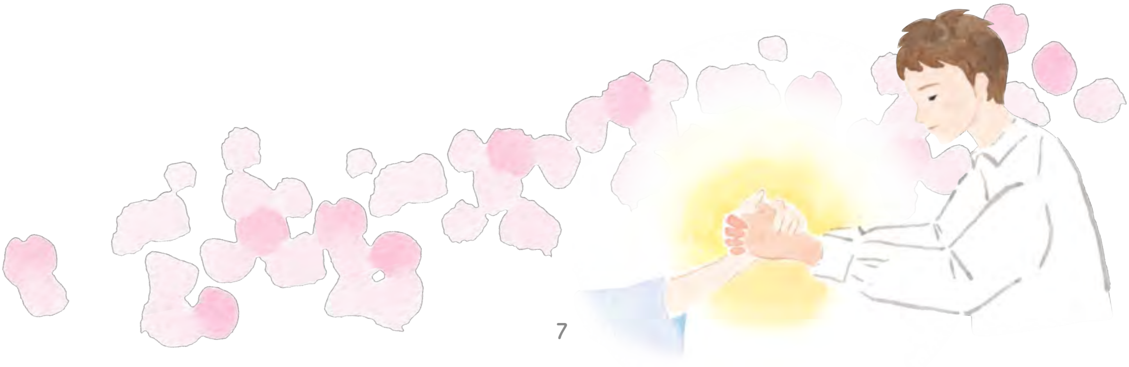

STEP
3

選択肢の特徴
（メリットとデメリット）を知る

治療の途中で、救命率が低くなり回復が難しくなった場合の治療の選択肢を比較してみましょう.

**すべての治療を受ける**	**延命効果を期待する治療をやめる**
**治療の内容**	
心身に大きな辛さや負担が伴う処置を受けても、できるだけ**長く生きる**ことを重視します. 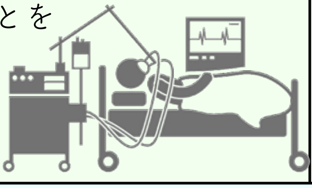	治療による延命効果を期待するよりも、できる限り**苦痛緩和やあなたらしい生き方**を大切にした治療を重視します. 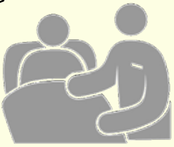
どちらを選択しても、入院中の不快感や苦痛はあなたが望む限り、薬物療法などで治療を続けられます.	
**生存率**	
延命することが可能になるかもしれません.<br><br>多臓器障害の方の1ヶ月の生存率は上昇傾向にありますが、**長期（1年以上）では37-74%**の**生存率**と報告があります.	病状に応じ心臓や呼吸が停止する可能性が高まったり、早まったりします.<br><br>生存率が低い場合、**望まない治療を受けずに済む**可能性があります.

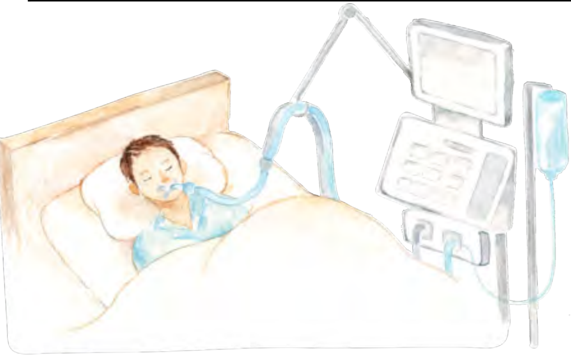

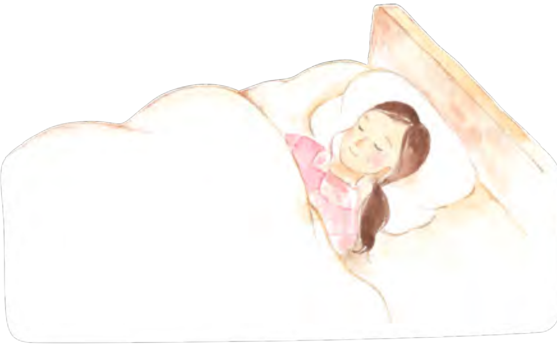

すべての治療を受ける	延命効果を期待する治療をやめる
**元の生活に戻ること**	
回復した場合、**70％**以上の人は日常生活の障害なく退院でき、元の生活へ復帰できる可能性があります. 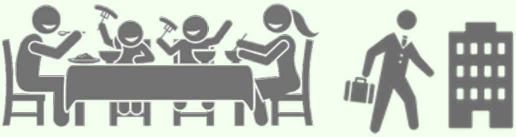	治療をやめたタイミングによりますが、日常生活活動は自分で行えない可能性があります. 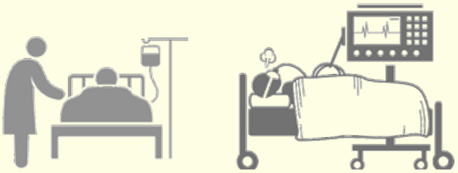
**認知・精神機能**	
回復した場合、**20-70％**の人は認知機能低下なく退院できる可能性があります. 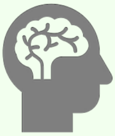<br><br>**8－57％**の人は治療後にも抑うつや不安症状が起こる可能性があり、その内、50％の人はこの症状が長く続く可能性があると報告されています.	治療をやめたタイミングによりますが、自分で考えたり伝えたりすることができない可能性が高いです.<br><br>痛みや辛さは薬でコントロールされるか、それを感じられる状態にない可能性があります.

**代理意思決定者の精神的影響**	
代理意思決定者の**10-80％**は、あなたがICUに入室していることで不安が増強したり抑うつ傾向になる可能性があります. 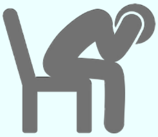	
あなたが回復すれば、不安やストレスが軽減する可能性があります.	あなたが亡くなると、不安やストレスは増強する可能性があります.
**医療費**	
ICUの入院期間や生命維持装置を装着する期間が長くなるほど、医療費は高くなります.	ICUの治療期間、治療内容により医療費がかかります. 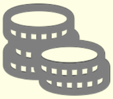

STEP 4 何を大事にして決めたいか明確にする

あなたの治療への希望は医学的な判断と同じくらい重要です.
あなたにとって大事なことは何か検討してみましょう.
あなたの気持ちに最も合う箇所にチェックを入れてみましょう.

チェック

**すべての治療を受ける**	**延命効果を期待する治療をやめる**

1. この判断おいて、あなたの**救命率**は、どのくらい重要ですか？

☐	☐	☐	☐	☐
全く重要でない		どちらでもない		とても重要

2. この判断において、あなたが**元の生活に戻れるか**どうかは、

☐	☐	☐	☐	☐
全く重要でない		どちらでもない		とても重要

3. あなたにとっての**重要な問題**は、何ですか？

**頭神経**	**心臓**	**肺**	**肝臓**	**腎臓**	**四肢**
☐	☐	☐	☐	☐	☐
意識があること、自分で判断できること等	補助循環装置は使いたくない、等	人工呼吸器の長期使用は嫌だ、等	補助的な器械は使いたくない、等	永久透析はしたくない、等	自分で歩いたり動くことができないのは嫌だ、等

すべての治療を受ける	延命効果を期待する治療をやめる

4. あなたにとって**その他の重要な項目**や気になる事項

・＿＿＿＿＿＿＿＿＿＿＿＿＿＿＿＿＿＿＿＿＿＿＿＿＿＿＿＿＿＿.

☐	☐	☐	☐	☐

全く重要でない　　どちらでもない　　とても重要

5. あなたには、「**どうしても受けたくない治療**」が、

☐ ない	☐ ある

内容：

6. この判断に関して、**医療費**がどの程度かかるかは、

☐	☐	☐	☐	☐

全く重要でない　　どちらでもない　　とても重要

7. この判断に関して、どんな時もあなたの意見を優先することは、

☐	☐	☐	☐	☐

全く重要でない　　どちらでもない　　とても重要

8. この判断に関して、あなたの信頼する人（代理意思決定者）の意見は、

☐	☐	☐	☐	☐

全く重要でない　　どちらでもない　　とても重要

9. この判断に関して、医療者の意見は、

☐	☐	☐	☐	☐

全く重要でない　　どちらでもない　　とても重要

STEP 5

決める

これまでのページで、あなたにとって何を大事にして決めたいかを考えました.
ここで、どのくらい**決める準備**ができたか見てみましょう.

チェック

当てはまるものにチェックを入れましょう.

あなたにとって最も良い選択だという自信がありますか？

☐ はい　☐ いいえ

あなたはそれぞれの選択肢の利益とリスク(危険性)を知っていますか？

☐ はい　☐ いいえ

あなたにとって、どの利益とリスク(危険性)が最も重要であるかはっきりしていますか？

☐ はい　☐ いいえ

選択するための十分な支援と助言がありますか？

☐ はい　☐ いいえ

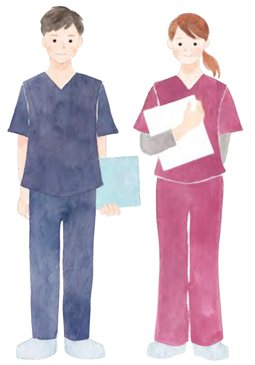

上記の4つのうち1つでも「いいえ」がついた場合には、
まだ決定の準備が十分整っていないかもしれません.
決める前に知りたいことや相談したいことは何か
ありますか？

決める

あなたが決めたことを記入しましょう.

　　　　年　　　　月　　　　日

☐ 救命率に関係なく、全ての治療を受ける

☐ 救命率がある程度低下したら、延命効果を期待する治療をやめる

私にとっての延命治療とは、

①医療者の判断として救命率が＿＿＿＿％程度と考えられる場合です.

②その他、＿＿＿＿＿＿＿＿＿＿＿＿＿＿＿＿＿＿です.

以下の治療は継続します（受けたい治療をチェック）

☐ 心肺蘇生　☐ 人工呼吸器の使用　☐ 補助循環装置の使用

☐ 透析　☐ 血圧維持のための薬の投与　☐ 輸液や栄養投与

☐ 輸血

考えたことを書いてみましょう

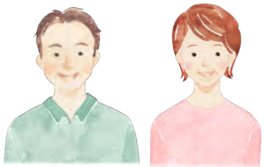

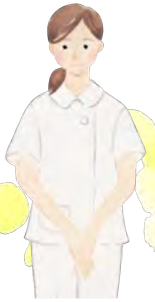

回復が難しくなった場合に受けたい治療を考えることは
気持ちが揺れたり、判断が難しいこともあります.
1度決めたら、2度と変えられないことはありません.

無事に手術を乗り越えられるように頑張りましょう.

参考にした海外のガイド

1 . Advance Care Planning: Should I Stop Treatment That Prolongs My Life? (Healthwise)
〔https://www.healthwise.net/ohridecisionaid/Content/StdDocument.aspx?DOCHWID=tu1430, 2020.3.4〕

引用文献/参考文献

1 Desai SV et al.（2011). Long-term complications of critical care. Crit Care Med, 39, 371-379.

2 Hervey MA et al. (2016). Postintensive Care Syndrome: Right Care. Right Now…and Later. Crit Care Med, 44, 381-358.

3 Winter BD et al.(2010). Long-term mortality and quality of life in sepsis: A systematic review. Crit Care Med. 38, 1276-1283

4 Coulter A et al.(2013). A systematic development process for patient decision aids. BMC Med Inform Decis Mak, 13 Suppl 2:S2.

5 中山和弘, 岩本貴. (編). (2012). 患者中心の意思決定支援　納得して決めるためのケア, 中央法規

作成者　：聖路加国際大学 山本加奈子（集中ケア認定看護師, 急性・重症患者看護専門看護師）
イラスト：taneko

作成日　：2020年 7月10日（このガイドは、定期的に情報や内容を見直し改訂を行います.）
改訂日　：2021年 3月31日
2019年度　文部科学研究費補助金(若手研究:19K19613 研究代表者: 山本加奈子)の助成により作成されたものです。

©2021, K Yamamoto. <無断複写・無断転用はご遠慮ください>
