# Supplementary material for: Implementation of advance care planning decision aids for patients undergoing high-risk surgery: a field-testing study
Source: BMC Palliat Care. 2022 Oct 12;21:179. doi: 10.1186/s12904-022-01068-2 (PMC9554854; doi:10.1186/s12904-022-01068-2)
Supplement: Supplementary file 3 — Additional file 3. [file 12904_2022_1068_MOESM3_ESM.docx]

**Additional files 3**

**Survey Contents**

**Patient**

| **No** | **Question** | **Answer** |
| --- | --- | --- |
| Pre-admission outpatient | | |
| 1 | Have you ever thought about what treatment you would like to receive during a life-threatening situation? | Yes, No |
| 2-1 | Which option do you choose?  1_ Do not communicate your ACP’s wishes to surrogate decision-makers and healthcare providers.  2_ Communicate your ACP's wishes to surrogate decision-makers and healthcare practitioners. |  |
| 2-2 | Sure test | Yes, No |
| 3-1 | Which option do you choose?  1_ Continue to receive all treatment regardless of survival rate  2_ Discontinue life-sustaining treatment when the survival rate decreases |  |
| 3-2 | Sure test | Yes, No |
| 4 | HADS | Likert scale |
| 5 | How old are you? |  |
| 6 | What is your sex. |  |
| 7 | Have you or your family ever been treated in an intensive care unit? |  |
| 8 | Is there a medical worker in your family? |  |
| 9 | What is your employment status. |  |
| On admission | | |
| 1 | How do you recognize the need to consider or discuss ACP before surgery? | NRS (0-10) |
| 2-1 | Which option do you choose?  1_ Do not communicate your ACP’s wishes to surrogate decision-makers and healthcare providers.  2_ Communicate your ACP's wishes to surrogate decision-makers and healthcare practitioners. |  |
| 2-2 | Sure test | Yes, No |
| 3-1 | Which option do you choose?  1_ Continue to receive all treatment regardless of survival rate  2_ Discontinue life-sustaining treatment when the survival rate decreases |  |
| 3-2 | Sure test | Yes, No |
| 4 | HADS | Likert scale |
| At first outpatient discharge | | |
| 1 | How do you recognize the need to consider or discuss ACP before surgery? | NRS (0-10) |
| 2-1 | Which option do you choose?  1_ Do not communicate your ACP’s wishes to surrogate decision-makers and healthcare providers.  2_ Communicate your ACP's wishes to surrogate decision-makers and healthcare practitioners. |  |
| 2-2 | Sure test | Yes, No |
| 3-1 | Which option do you choose?  1_ Continue to receive all treatment regardless of survival rate  2_ Discontinue life-sustaining treatment when the survival rate decreases |  |
| 3-2 | Sure test | Yes, No |
| 4 | HADS | Likert scale |
| 5 | How often did you read the PtDAs (PtDA_A, PtDA_B)? | Likert scale |
| 6 | How is the amount of information given by the PtDAs (PtDA_A, PtDA_B)? | Likert scale |
| 7 | Were the PtDAs (PtDA_A, PtDA_B) easy to understand? | Likert scale |
| 8 | Do you want to recommend these PtDAs to other patients? | Likert scale |
| 9 | Did you get information from sources other than these PtDAs? |  |

**Family**

| **No** | **Question** | **Answer** |
| --- | --- | --- |
| Pre-admission outpatient | | |
| 1 | Have you ever had a discussion with a patient about ACP? | Yes, No |
| 2 | Which option do you think your family (patient) will choose?  1_ Continue to receive all treatment regardless of survival rate  2_ Discontinue life-sustaining treatment when the survival rate decreases |  |
| 3 | How confident are you that you are able to make the same choices as the patient? | NRS (0-10) |
| 4 | HADS | Likert scale |
| 5 | How old are you? |  |
| 6 | What is your sex. |  |
| 7 | What is the relationship between you and the patient? |  |
| 8 | Is there a medical worker in your family? |  |
| 9 | What is your employment status? |  |
| 10 | Have you ever been associated with end-of-life care decisions? |  |
| On admission | | |
| 1 | Did you discuss ACP with the patient and the healthcare providers before surgery? |  |
| 2 | Which option do you think your family (patient) will choose?  1_ Continue to receive all treatment regardless of survival rate  2_ Discontinue life-sustaining treatment when the survival rate decreases |  |
| 3 | How confident are you that you are able to make the same choices as the patient? | NRS (0-10) |
| 4 | How do you recognize the need to discuss ACP with patients before surgery? | NRS (0-10) |
| 5 | HADS | Likert scale |
| At first outpatient discharge | | |
| 1 | Did you discuss ACP with the patient or the healthcare provider after the patient was discharged? | NRS (0-10) |
| 2 | Which option do you think your family (patient) will choose?  1_ Continue to receive all treatment regardless of survival rate  2_ Discontinue life-sustaining treatment when the survival rate decreases |  |
| 3 | How confident are you that you are able to make the same choices as the patient? | NRS (0-10) |
| 4 | How do you recognize the need to discuss ACP with patients before surgery? | NRS (0-10) |
| 5 | HADS | Likert scale |
